# A complete theoretical framework for inferring horizontal gene transfers using partial order sets

**DOI:** 10.1371/journal.pone.0281824

**Published:** 2023-03-24

**Authors:** Nahla A. Belal, Lenwood S. Heath

**Affiliations:** 1 Department of Computer Science, Arab Academy for Science, Technology, and Maritime Transport, Alexandria, Egypt; 2 Department of Computer Science, Virginia Tech, Blacksburg, VA, United States of America; Universite du Quebec a Montreal, CANADA

## Abstract

We present a method for detecting horizontal gene transfer (HGT) using partial orders (posets). The method requires a poset for each species/gene pair, where we have a set of species *S*, and a set of genes *G*. Given the posets, the method constructs a phylogenetic tree that is compatible with the set of posets; this is done for each gene. Also, the set of posets can be derived from the tree. The trees constructed for each gene are then compared and tested for contradicting information, where a contradiction suggests HGT.

## Introduction

Most work in evolutionary genomics has focused on vertical gene transfer from one species to a lineal descendant. Much recent work has been directed towards the phenomenon of horizontal gene transfer (HGT) [1]. Because of the impact of HGTs on the ecological and pathogenic character of genomes, algorithms are sought that can computationally determine which genes of a given genome are products of HGT events. Numerous strategies have employed nucleotide composition of coding sequences to predict HGT. Previous methods marked the genes with a typical G + C content. Other methods used codon usage patterns to predict HGT. Also, many models used nucleotide patterns for genomic signature, these models have been analyzed using sliding windows, Bayesian classifiers, Markov models, and support vector machines. While no previous work uses partial orders to investigate HGT, we do summarize computational research for detecting HGT in the later Related Literature section.

**Fig 1:**
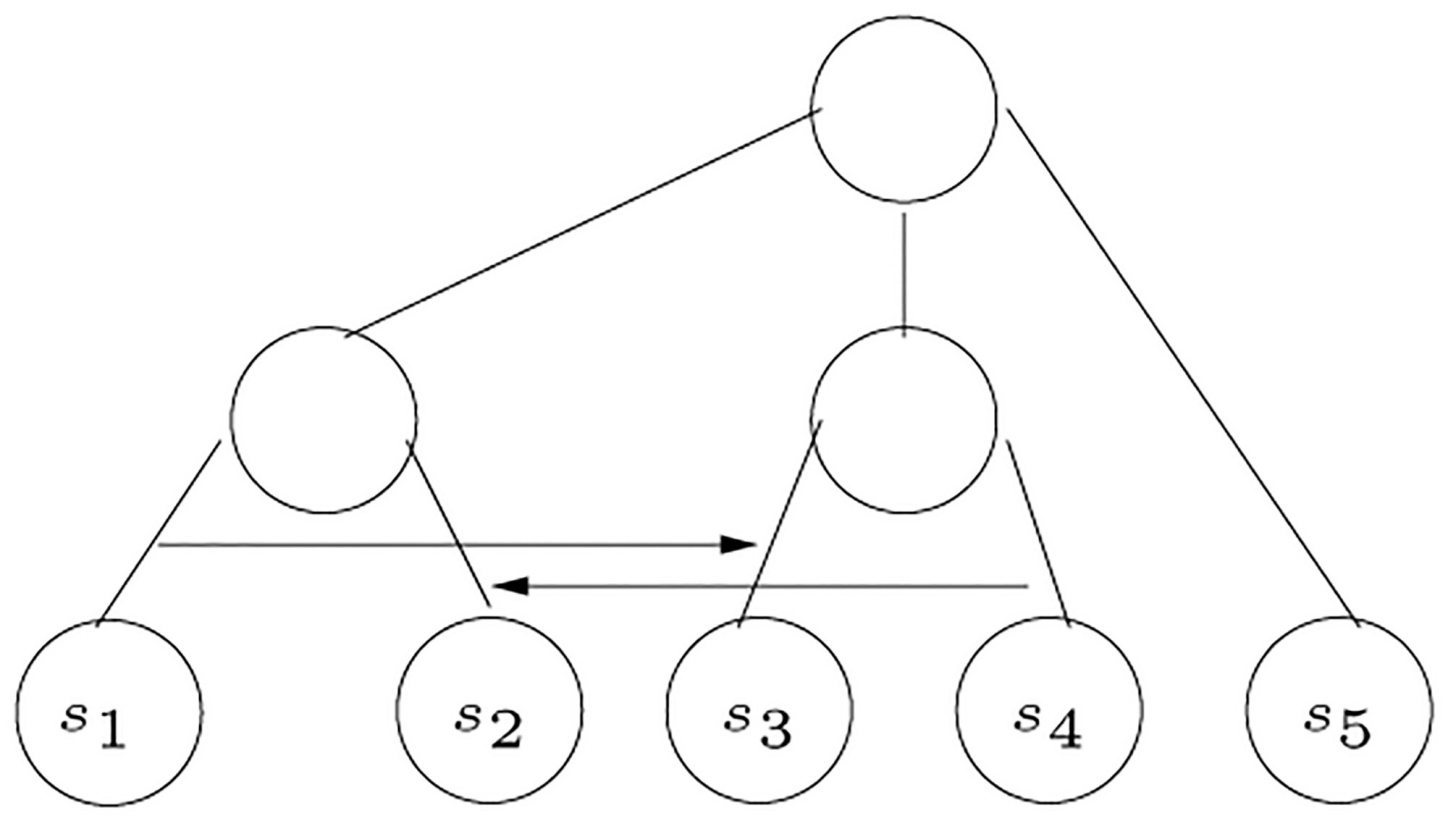
The possible HGT events for the example in Fig 34.

Suppose that we have complete, annotated genomes for *m* species. Further, suppose that we have selected a set of *n* genes, from some reference genome or otherwise, for analysis. If we know the relative distances between each pair of species per gene, then we have a set of partial orders defining the relative relationship among species that can be used to identify which genes are candidates for HGT. Given a poset for each gene, a tree corresponding to that gene is constructed; different trees suggest genes that are candidates for HGT. Once HGT is indicated, additional time-related information can be brought to bear to determine the relative order of events and to establish direction. In fact, our algorithm predicts direction as illustrated in Fig 1.

Suppose that we have complete, annotated genomes for species *s*_1_, *s*_2_, …, *s*_*m*_. Further, suppose that we have selected a set of genes, from some reference genome or otherwise, for analysis. Let those genes be *g*_1_, *g*_2_, …, *g*_*n*_. Standard methods for obtaining the set of genes, such as the one in Lake and Rivera [2], can be followed. BLASTing gene *g*_*k*_ in species *s*_*i*_ against a database of genes from all *m* species, we obtain a bit score *B*(*g*_*k*_; *s*_*i*_, *s*_*j*_) of a best alignment of that gene against the same gene in species *s*_*j*_. If *g*_*k*_ is not found in *s*_*j*_, then set *B*(*g*_*k*_; *s*_*i*_, *s*_*j*_) = 0. In general, the higher *B*(*g*_*k*_; *s*_*i*_, *s*_*j*_) is, the better the match between gene *g*_*k*_ in species *s*_*i*_ and gene *g*_*k*_ in species *s*_*j*_. There is no need to take special notice of an absent gene, since *B*(*g*_*k*_; *s*_*i*_, *s*_*j*_) = 0 is a meaningful substitute for a Boolean value representing presence or absence of a gene.

There is another quantity associated with the (*g*_*k*_, *s*_*i*_, *s*_*j*_) triple. Define *T*(*g*_*k*_; *s*_*i*_, *s*_*j*_) to be the true evolutionary distance, this means what actually happened during the process of gene evolution, in time between the *g*_*k*_ gene of *s*_*i*_ and the *g*_*k*_ gene of *s*_*j*_. For example, if the most recent common ancestor of the two genes existed 20 million years ago, then *T*(*g*_*k*_; *s*_*i*_, *s*_*j*_) is 40 million years. While these *T*(*g*_*k*_; *s*_*i*_, *s*_*j*_) values cannot be measured directly, either absolute or relative values for times can be estimated using probabilistic models.

The *B*(*g*_*k*_; *s*_*i*_, *s*_*j*_) values are not random. In fact, a ranking of the *B*(*g*_*k*_; *s*_*i*_, *s*_*j*_) values for 1 ≤ *j* ≤ *m* should roughly match a ranking of the *T*(*g*_*k*_; *s*_*i*_, *s*_*j*_) values from the *s*_*i*_ gene *g*_*k*_ to all the other *g*_*k*_’s. In the absence of HGT or other horizontal evolutionary events, we must have *T*(*g*_*k*_; *s*_*i*_, *s*_*j*_) = *T*(*g*_ℓ_; *s*_*i*_, *s*_*j*_) for every pair of genes *g*_*k*_ and *g*_ℓ_. Therefore, we expect that the rankings of the *B*(*g*_*k*_; *s*_*i*_, *s*_*j*_) and *B*(*g*_ℓ_; *s*_*i*_, *s*_*j*_) values will be similar in ways we want to explore. And, under reasonable assumptions, the distribution of relative distances should be consistent with predictions of coalescent theory. In particular, as evolutionary distances increase, there will typically be multiple genes that have the same *T* value from the *g*_*k*_ gene in species *s*_*i*_. Moreover, the probability that two evolutionary events occur at the same instance in time is 0.

In the presence of horizontal evolutionary events, the patterns of rankings of the *B* and *T* values will be different for different genes, depending on which horizontal events each gene is involved in. Two genes that are involved in exactly the same horizontal events will have identical patterns in their *T* values and similar patterns in their *B* values.

If we use the rankings of the *B* values as an approximate substitute for the rankings of the unknown *T* values, then the rankings can be compared and clustered to identify groups of genes that participated in the same horizontal events. Fix a gene *g*_*k*_. Then there is a gene *g*_*k*_ tree that represents the true evolutionary history of the *g*_*k*_’s in all the species. It is rooted at the most recent common ancestor of the *m* species. Our first goal is to define a computational problem to achieve this clustering and to design an efficient algorithm to solve the problem. In the following, proofs of results are elaborated. Note that Belal and Heath [3] is an earlier five-page announcement of these results.

## Definitions

For a rooted (directed) tree *T*, let *R*(*T*) be the root of *T*, let *I*(*T*) be the set of internal nodes of *T*, and let *L*(*T*) be the set of leaves of *T*.

**Fig 2:**
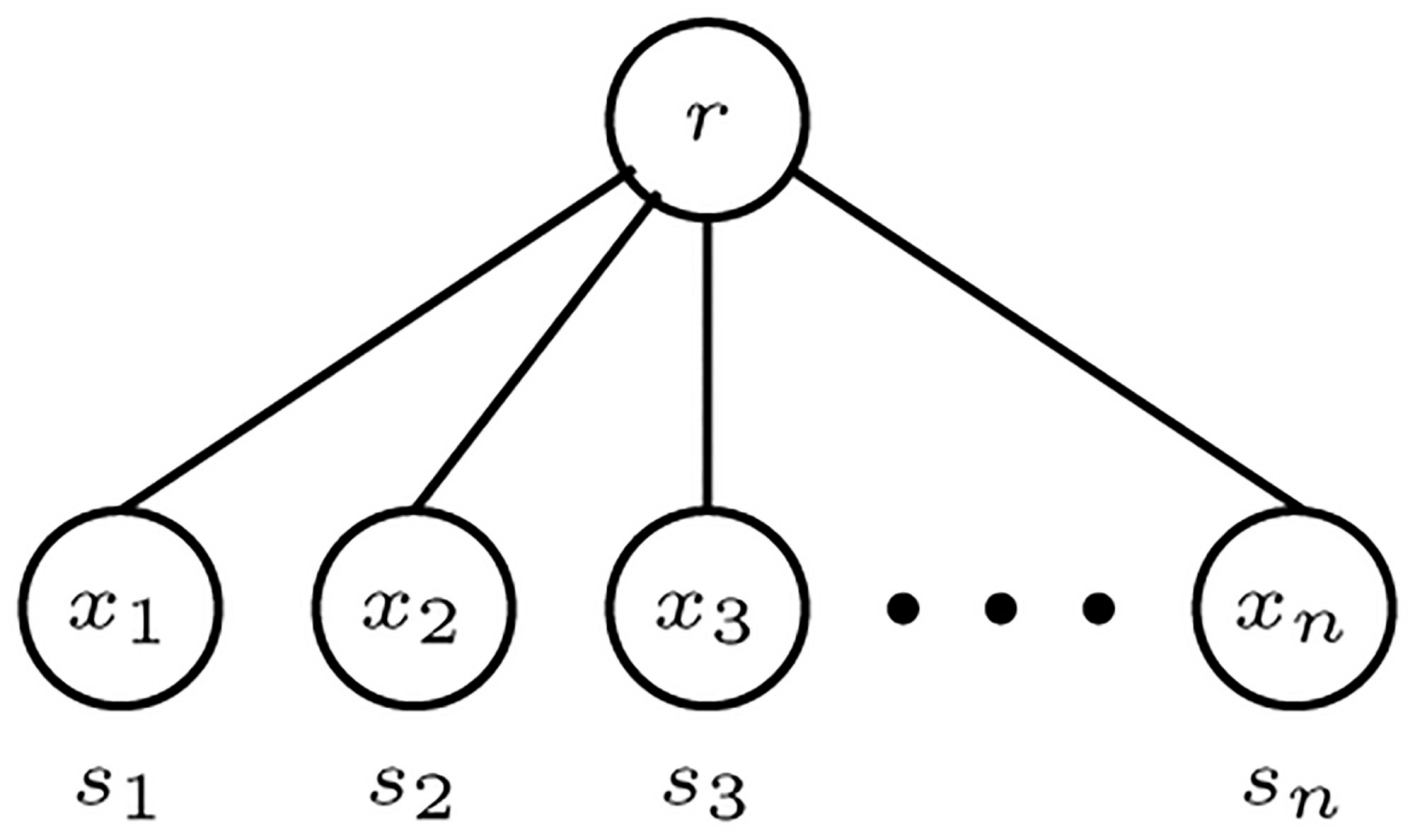
A trivial non-binary *S*-tree with a minimum number of nodes and no evolutionary assumptions [3].

**Fig 3:**
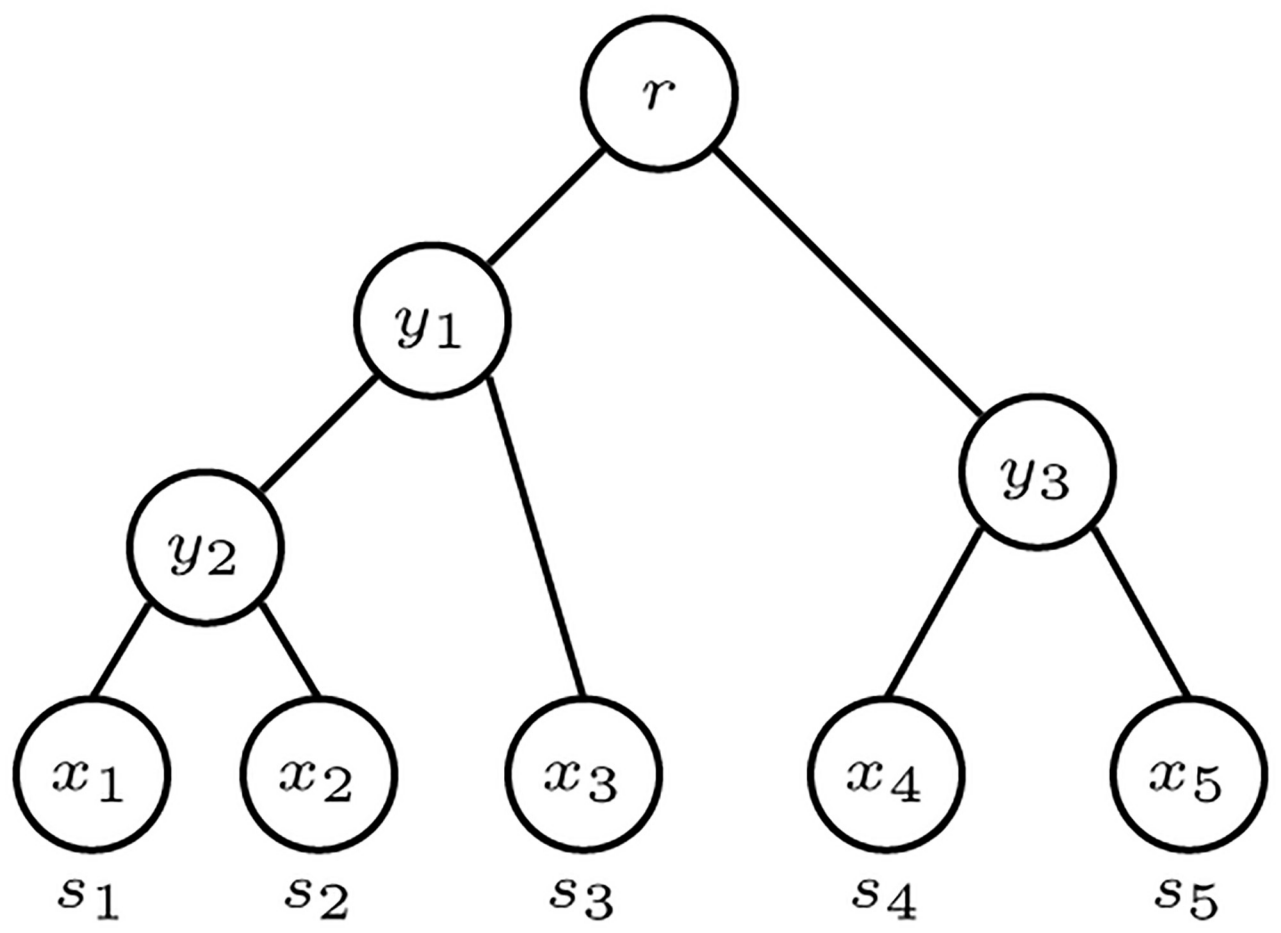
An evolutionary *S*-tree with 5 taxa [3].

Let *S* be a finite set of species. An *S*-tree *T* = (*V*, *E*) is a rooted tree such that every internal node has outdegree at least two and a bijective *labeling function* *λ*: *L*(*T*) → *S*. In particular, every *S*-tree has precisely |*S*| leaves. Fig 2 illustrates an *S*-tree for the case *n* ≥ 2, where there is only one internal node, the root *r* = *R*(*T*). There are *n* leaves *x*_1_, *x*_2_, …, *x*_*n*_ and *λ*(*x*_*i*_) = *s*_*i*_. If every internal node of *T* has outdegree exactly two, then *T* is an *evolutionary tree*. Fig 3 illustrates an evolutionary tree on five species.

Let *T* = (*V*, *E*) be an *S*-tree. Let *u* ∈ *V*. The subtree rooted at *u* is *T*(*u*). The *species set S*(*u*) for *u* is the set of leaf labels in *T*(*u*).

**Fig 4:**
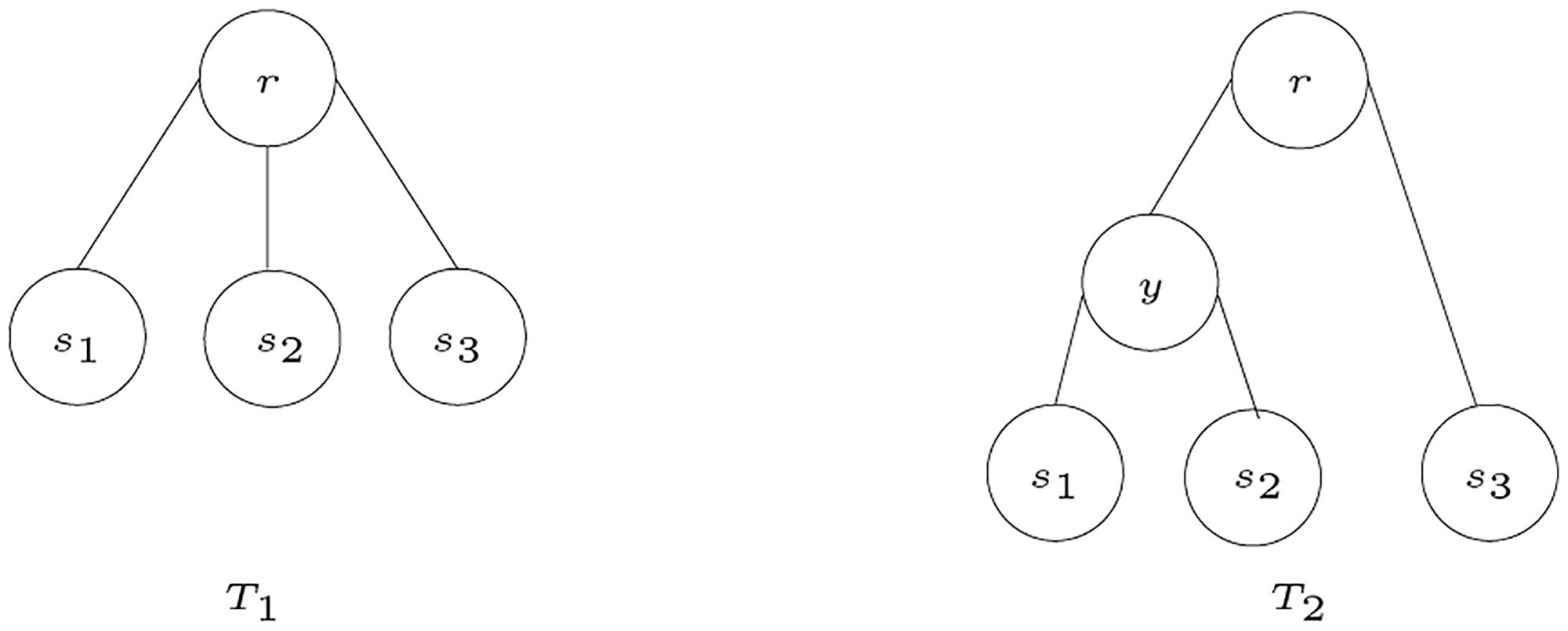
Refinement of *T*_1_ to *T*_2_ [3].

Let *T* be an *S*-tree with an internal node *x* that has three or more children. A *refinement step* (on *T* at *x*) adds an internal node *y* to the tree *T*, where *y* is the parent of a proper subset of the children of *x* and *y* is a new child of *x*. An *S*-tree *T*′ is a *refinement* of *T* if *T*′ can be obtained by performing zero or more refinement steps on *T*. For example, in Fig 4, *T*_2_ is a refinement of *T*_1_ by a refinement step on *T*_1_ at *r*. The refinement step applied adds one internal node *y*, which is the parent of *s*_1_ and *s*_2_ in *T*_2_; *y* and *s*_3_ are the direct children of *r* in *T*_2_.

Let *X* = {*X*_1_, *X*_2_} and *Y* = {*Y*_1_, *Y*_2_} be two partitions of *S*. Call such partitions with two elements each *2-partitions*. Note that the deletion of an edge from an *S*-tree induces two connected subtrees and, hence, a 2-partition of *S*. *X* and *Y* are *contradicting partitions* if there exist four species *s*_1_, *s*_2_, *s*_3_, *s*_4_ such that *s*_1_, *s*_2_ ∈ *X*_1_, *s*_3_, *s*_4_ ∈ *X*_2_, *s*_1_, *s*_3_ ∈ *Y*_1_, and *s*_2_, *s*_4_ ∈ *Y*_2_. Two *S*-trees *T*_1_ and *T*_2_ are *contradictory* if their exists an edge in *T*_1_ and an edge in *T*_2_ such that their induced 2-partitions are contradicting.

Let *u*, *v* ∈ *L*(*T*), for some *S*-tree *T*. The *most recent common ancestor* MRCA(*u*, *v*) of *u* and *v* is the node *w* that is a common ancestor of *u* and *v* such that *T*(*w*) is the smallest rooted subtree in *T* containing both *u* and *v*.

A *partial order* is a binary relation ≤ over a set *S* that is reflexive, antisymmetric, and transitive, i.e., for all *a*, *b*, *c* ∈ *S*, we have that

- *a* ≤ *a* (reflexivity);
- if *a* ≤ *b* and *b* ≤ *a* then *a* = *b* (antisymmetry); and
- if *a* ≤ *b* and *b* ≤ *c* then *a* ≤ *c* (transitivity).

**Fig 5:**
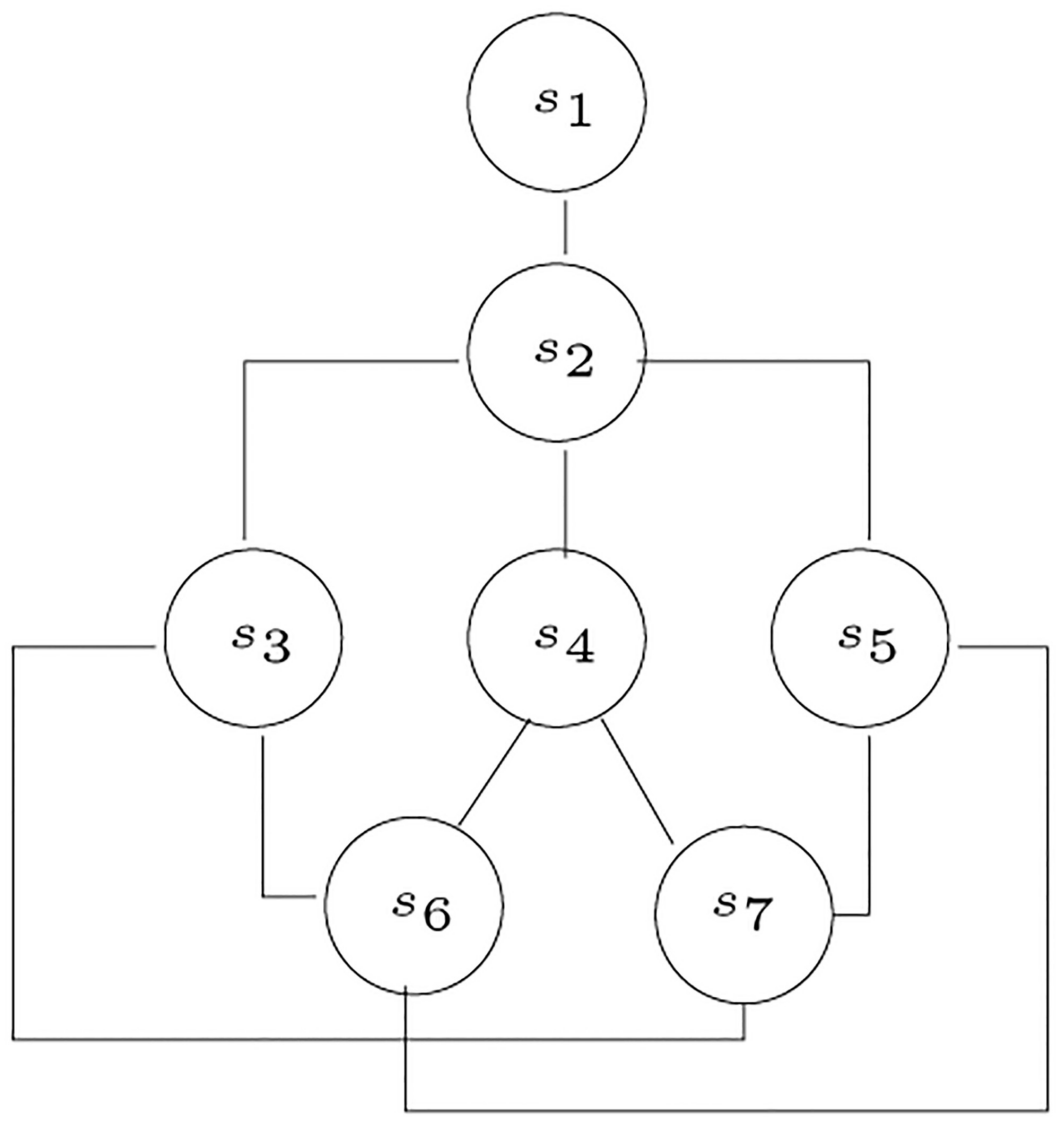
An example of a Hasse diagram.

A set with a partial order is a *partially ordered set* or a *poset*. If (*S*, ≤) is a poset and *a*, *b* ∈ *S*, then *a* < *b* if and only if *a* ≤ *b* and *a* ≠ *b*. Note that *a* < *b* is transitive. The directed graph *G* = (*S*, <) is clearly a directed acyclic graph (DAG). The *transitive reduction* of *G* is the DAG on node set *S* that contains those edges (*a*, *b*) such that there is no *c* ∈ *S* satisfying *a* < *c* < *b*. A *Hasse diagram* of < (which is also a Hasse diagram of ≤) is a drawing of the transitive reduction of (*S*, <) such that no arrows are included. An example of a Hasse diagram is shown in Fig 5. The diagram shown corresponds to the following poset:

$$\begin{array}{ccc} P & = & \{\left(s_{1},s_{2}\right),\left(s_{1},s_{3}\right),\left(s_{1},s_{4}\right),\left(s_{1},s_{5}\right),\left(s_{1},s_{6}\right),\left(s_{1},s_{7}\right),\\ & & \left(s_{2},s_{3}\right),\left(s_{2},s_{4}\right),\left(s_{2},s_{5}\right),\left(s_{2},s_{6}\right),\left(s_{2},s_{7}\right),\\ & & \left(s_{3},s_{6}\right),\left(s_{3},s_{7}\right),\left(s_{4},s_{6}\right),\left(s_{4},s_{7}\right),\left(s_{5},s_{6}\right),\left(s_{5},s_{7}\right)\}. \end{array}$$

Let *s*_*i*_ ∈ *S* be a species. An *s_i_-poset* *P* = (*S*, ≤_*i*_) is a poset with the property that, for every *s*_*j*_ ∈ *S*, we have *s*_*i*_ ≤_*i*_ *s*_*j*_. In other words, *s*_*i*_ is the unique minimum element of *P*.

**Fig 6:**
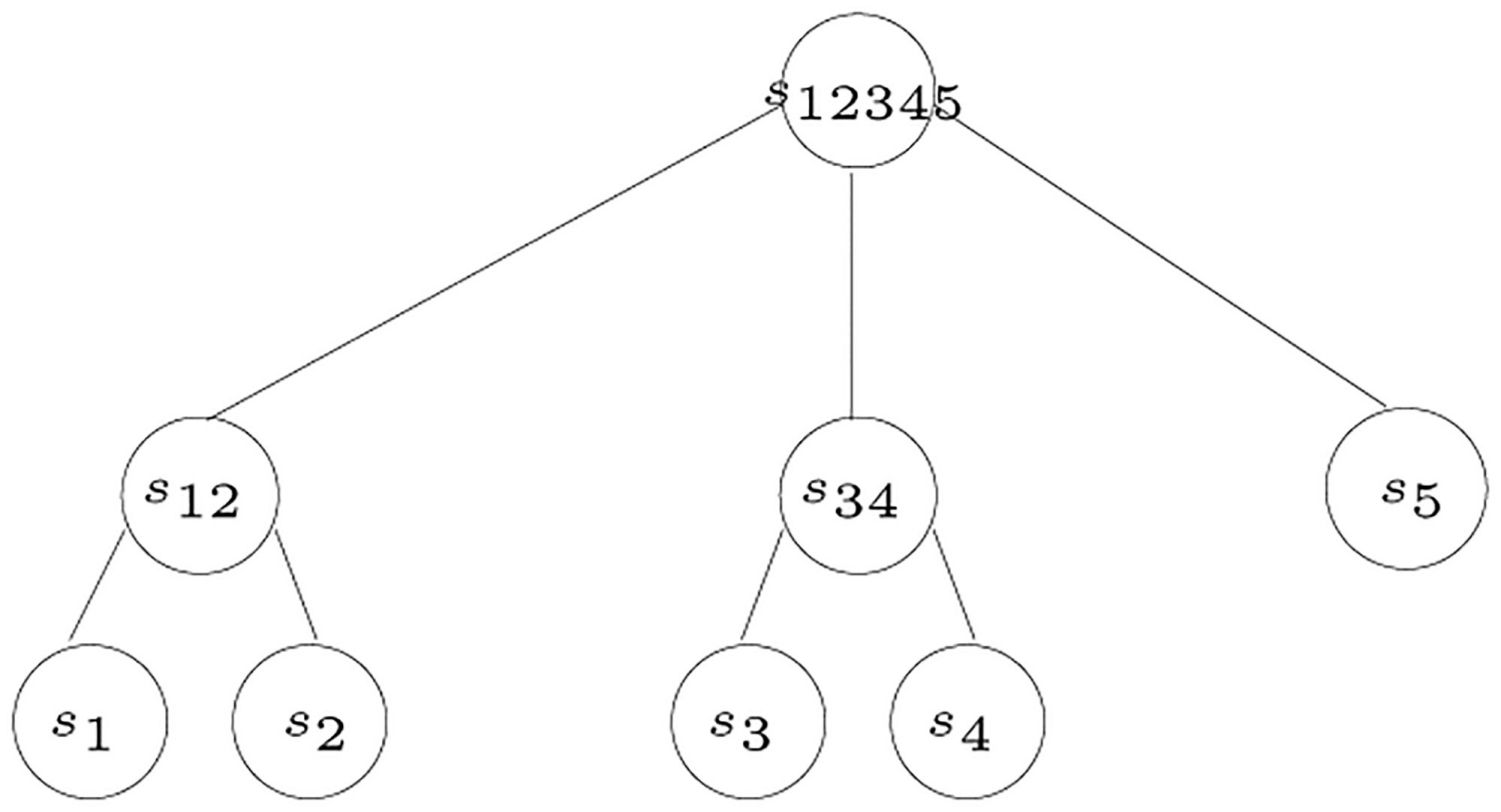
An example of a tree to test compatibility with posets [3].

**Fig 7:**
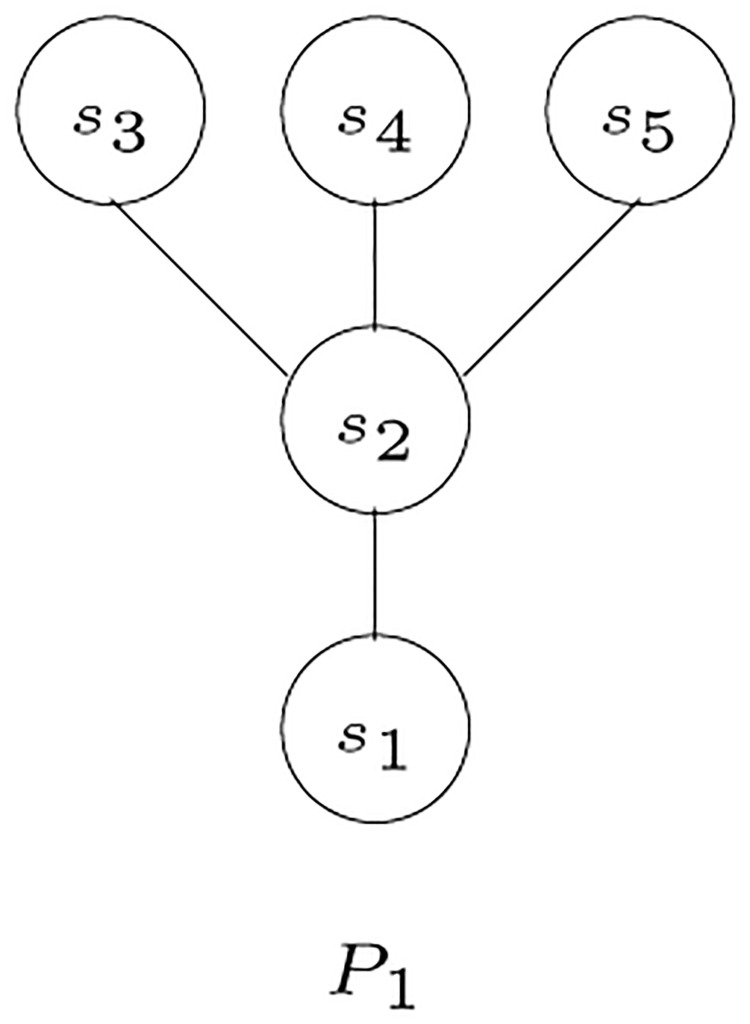
An example of a poset compatible with the tree in Fig 6 [3].

**Fig 8:**
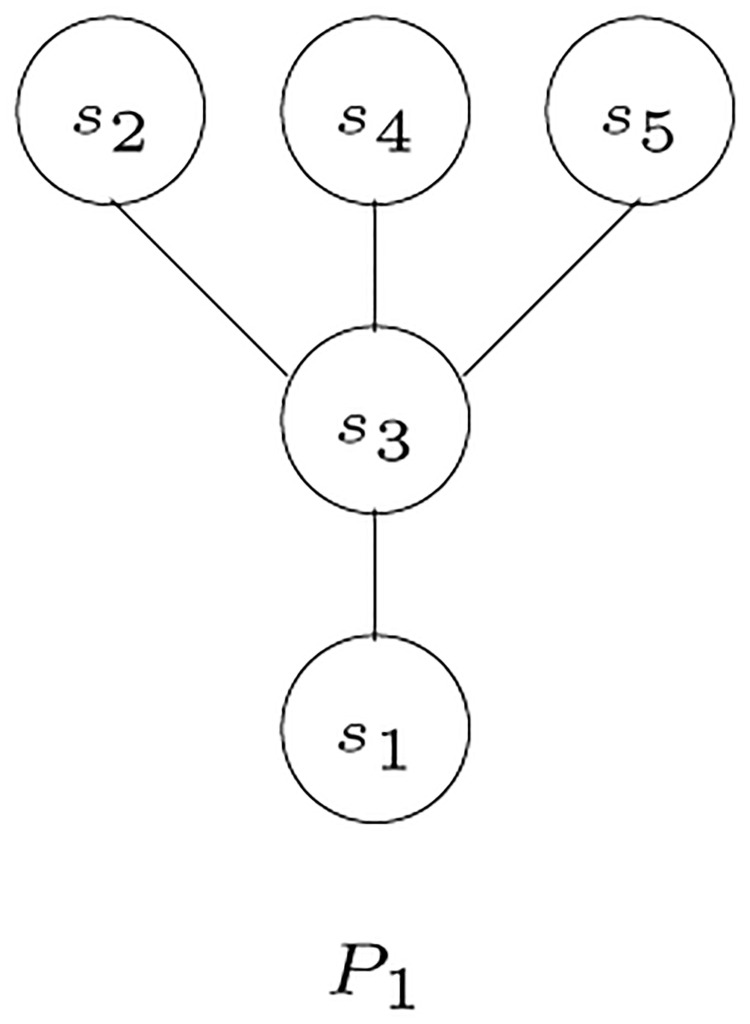
An example of a poset incompatible with the tree in Fig 6 [3].

The *s*_*i*_-poset *P*_*i*_ = (*S*, ≤_*i*_) is *compatible* with *S*-tree *T* if, for all distinct triples *x*, *y*, *z* ∈ *L*(*T*) such that *λ*(*x*) = *s*_*i*_, *λ*(*y*) = *s*_*j*_, and *λ*(*z*) = *s*_*k*_ and such that *s*_*j*_≤_*i*_*s*_*k*_, then we have the shortest path from either of *x* or *y* to *z* passes through MRCA (*x*, *y*). Given the tree shown in Figs 6, 7 shows an example of a poset that is compatible with the given tree, while Fig 8 shows an incompatible poset, where the poset indicates that *s*_3_ is the closest species to *s*_1_, while, in the tree, the closest species to *s*_1_ is *s*_2_.

Let $P=\{P_{1},P_{2},\ldots ,P_{n}|P_{i}\;\text{is}\;\text{an}\;s_{i}-\text{poset}\}$ be a set of posets. P is *consistent* if, for all posets *P*_*i*_, *P*_*j*_ ∈ *P*, whenever *s*_*j*_≤_*i*_*s*_*k*_, then *s*_*i*_≤_*j*_*s*_*k*_. For example, let *P*_1_ = {(*s*_1_, *s*_2_), (*s*_1_, *s*_3_), (*s*_2_, *s*_3_)}, *P*_2_ = {(*s*_2_, *s*_1_), (*s*_2_, *s*_3_), (*s*_1_, *s*_3_)}, and *P*_3_ = {(*s*_3_, *s*_1_), (*s*_3_, *s*_2_)}. Then, {*P*_1_, *P*_2_, *P*_3_} is consistent. However, if *P*_4_ = {(*s*_3_, *s*_1_), (*s*_3_, *s*_2_), (*s*_1_, *s*_2_)}, then {*P*_1_, *P*_2_, *P*_4_} is inconsistent, since *P*_1_ and *P*_2_ indicate that *s*_1_ and *s*_2_ are closer to each other than to *s*_3_, while *P*_4_ indicates that *s*_1_ is closer to *s*_3_ than to *s*_2_.

## Related literature

Among the methods for detecting HGT addressed by many researchers is conditioned reconstruction. Conditioned reconstruction (CR) is a phylogenetic technique that utilizes gene absence/presence data to reconstruct phylogenetic relationships [4]. CR [2], compares a genomic sequence to another and according to whether a gene ortholog is present or absent supplies a P or A character state. The probability of a state transition is analyzed using Markov models. Given two genes, *X* and *Y*, four patterns are possible, PP, PA, AP, and AA. Many questions were raised on how to count the pattern AA. How can one identify genes that are missing from both genomes *X* and *Y*. To solve this problem, CR uses a conditioning genome, as a reference to which genes to be considered. A gene has to be present in both the conditioning genome and the genome being coded, in order to be considered present. An absent gene is present in the conditioning genome and absent from the genome under study. The conditioning genome has a big effect on the results obtained, as it represents the full set of orthologous genes coded during matrix development. In our approach, we avoid building our results on a conditioning genome, or any other input that would bias our results. However, the approach we present is similar to CR in the problem addressed and the use of information about all genes in the genomes. Bailey et al. [4] argue that CR cannot be used to distinguish between HGT and genome fusion. They suggest some refinements that make CR perform better. Bapteste and Walsh [5] question the ring of life hypothesis of Lake and Rivera [2]. They claim that it is not possible to reconstruct the ring of life in the presence of HGT. Bapteste and Walsh [5] see that the conditioning genome (CG) is more a tool than a biological concept, this genome can exist anywhere in the tree of life and can not be used in evolutionary reconstruction. See Belal [6] for additional discussion of CR. Related methods are found in [7–11].

Other methods for detecting horizontal gene transfer are proposed by multiple researchers. Podell and Gaasterland [12] present the DarkHorse method for detecting HGT. They defined the LPI, lineage probability index, to measure HGT and species closeness. This measure relies on lineage key terms. The higher the LPI score for an organism, the closer it is to the query (reference) genome. Groups of closely related organisms, have similar LPI scores. Xiang et al. [13] apply DarkHorse in analyzing the evolutionary relationship between Microsporidia and Fungi.

Moreover, phylogenetic reconstruction research contributed in solving many evolutionary problems. Nakhleh et al. [14] present a method for reconstructing phylogenetic networks using maximum parsimony. Their method is then studied and applied in [15]. Other network-based methods are found in [16–20]. For example, Cardona, Pons, and Rosselló [17] investigate LGT (lateral gene transfer) networks that combine a principal rooted subtree with a set of additional edges representing LGT. They present an efficient algorithm for constructing an LGT network from a set of phylogenetic trees.

Snir and Trifonov [21] present a method for detecting HGT. Their algorithm takes two genomes with their lengths and calculates the expectancy of each identical region’s length to obtain a measure of confidence as to exceptional similarity. Abby et al. [22] present a program called Prunier for the detection of HGT. The program searches for a maximum statistical agreement forest between a gene tree and a reference tree. Adato et al. [23] provide an algorithm for detecting HGT based on gene synteny and the concept of constant relative mutability. Scornavacca et al. [24] provide an algorithm for detecting HGT in some alternative cases. Sanchez-Soto et al. [25] introduce the algorithm ShadowCaster for HGT detection in prokaryotes.

Some researchers combine HGT with other evolutionary phenomena. Bansal et al. [26] develop the tool RANGER-DTL to detect gene duplication, transfer, and loss. Van Iersel et al. [27] develop a polynomial-time algorithm for some cases of HGT detection. Hasic and Tannier [28] present NP-hard cases for HGT detection.

In addition to the above, there are a number of theoretical approaches to problems related to HGT transfer: [28–31]. These are typically about mathematically-oriented methodologies for reconstructing a species tree or reconciling gene and species trees.

Also worth discussing, is reticulate evolution. According to [32], there are numerous reticulations among related species, especially in insects, vertebrates, microbes, and plants. In [33], extensions of Wayne Maddison’s approach are presented for reconstructing reticulate evolution that result from horizontal transfer or hybrid speciation. Two polynomial time algorithms are presented and outperform both NeighborNet and Maddison’s method. Moreover, [34] gives a review of the mathematical techniques used to construct phytogenies and reticulate evolution. Different methods are discussed, among which are distance-based, maximum parsimony, and maximum likelihood methods. In [35], the problem of approximating a dissimilarity matrix using a reticulogram is discussed, where it is obtained by adding edges an additive tree which implies improving the approximation of the dissimilarity matrix. As stated in [36], Horizontal gene transfer (HGT) is one of the most important events in evolution and they describe a new polynomial-time algorithm to infer HGT events. The algorithm uses least squares (LS), Robinson and Foulds (RF) distance, quartet distance (QD), and bipartition dissimilarity (BD). The results show that bipartition dissimilarity gives the best results.

Also, in [37] a novel heuristic technique for HGT detetction was employed for and tested on both simulated and real data. The technique was found to provide a greater sensitivity than other HGT techniques. The proposed technique also considers the lengths of the genes being transferred.

In [38] a number of operons have been identified experimentally by sequence similarity analysis and then by phylogenetic analysis. Many occurrences of horizontal transfer of entire operons were detected.

Mosaic genes have been discussed in [39]. A mosaic gene is composed of alternating sequence polymorphisms either belonging to the host original allele or derived from the integrated donor DNA. In this paper, the authors propose a method for detecting partial HGT events and related intragenic recombination giving rise to the formation of mosaic genes.

## Constructing an *S*-tree from a set of posets

Recall the definition of compatible from the Definitions Section. The *s*_*i*_-poset *P*_*i*_ = (*S*, ≤_*i*_) is *compatible* with *S*-tree *T* if, for all distinct triples *x*, *y*, *z* ∈ *L*(*T*) such that *λ*(*x*) = *s*_*i*_, *λ*(*y*) = *s*_*j*_, and *λ*(*z*) = *s*_*k*_ and such that *s*_*j*_≤_*i*_*s*_*k*_, then we have the shortest path from either of *x* or *y* to *z* passes through MRCA (*x*, *y*).

The problem of constructing a tree is defined as follows:

Compatible Tree Construction
INSTANCE: Set *S* = {*s*_1_, *s*_2_, …, *s*_*n*_} of *n* taxa; for 1 ≤ *i* ≤ *n*, an *s*_*i*_-poset *P*_*i*_ = (*S*, ≤_*i*_).
SOLUTION: An *S*-tree *T* compatible with *P*_1_, *P*_2_, …, *P*_*n*_, if one exists.

**Theorem 1**. *Let* P *be a set of posets that is compatible with an S-tree T. Let T′ be a refinement of T. Then* P *is compatible with T′*.

*Proof*. The proof is by induction on the number of refinement steps, *k*, to obtain *T*′ from *T*. For the base case of the induction, assume that *k* = 0. Then *T*′ = *T*, and, therefore, P is clearly compatible with *T*′. Now assume that *k* ≥ 1 and that the result holds for *k* − 1 refinement steps. Then there exists an *S*-tree *T*^′′^ such that *T*^′′^ is obtained by *k* − 1 refinement steps from *T* and *T*′ is obtained from *T*^′′^ in one refinement step. Let *u* in *T*^′′^ have children *v*_1_, *v*_2_, …, *v*_*p*_ such that in *T*′ there is a new node *w* that is a child of *u* with children *v*_1_, *v*_2_, …, *v*_*q*_, where *u* retains children *v*_*q* + 1_, …, *v*_*p*_ in *T*′. Note that *q* ≥ 2 and *p* − *q* ≥ 1. Therefore, for P to be compatible with *T*′, the compatibility condition must hold, and that is:

- For all distinct triples *x*, *y*, *z* ∈ *L*(*T*) such that *λ*(*x*) = *s*_*i*_, *λ*(*y*) = *s*_*j*_, and *λ*(*z*) = *s*_*k*_ and such that *s*_*j*_≤_*i*_*s*_*k*_, then there is a shortest path from either of *x* or *y* to *z* passing through MRCA (*x*, *y*).

By applying the compatibility condition to *T*^′′^, the cases for *x*, *y*, and *z* are as follows:

- *x* ∈ *v*_1_, *v*_2_, …, *v*_*p*_ or *y* ∈ *v*_1_, *v*_2_, …, *v*_*p*_. Since *s*_*j*_≤_*i*_*s*_*k*_, therefore, there exists an MRCA for *x* and *y*. Let MRCA (*x*, *y*) be *q*. Therefore, the shortest path from either of *x* or *y* to *z* passes through *q*.
- *x*, *y* ∈ *v*_1_, *v*_2_, …, *v*_*p*_. Therefore, MRCA (*x*, *y*) is *u*, and the shortest path from either of *x* or *y* to *z* passes through *u*.
- *x*, *y* ∉ *v*_1_, *v*_2_, …, *v*_*p*_. Since, *s*_*j*_≤_*i*_*s*_*k*_, therefore, there exists an MRCA for *x* and *y* such that the shortest path from either of *x* or *y* to *z* passes through the MRCA (*x*, *y*).

Similarly, by applying the compatibility condition to *T*′, the cases for *x*, *y*, and *z* are as follows:

- *x*, *y* ∈ *v*_1_, *v*_2_, …, *v*_*q*_. Therefore, MRCA (*x*, *y*) is *w*, and the shortest path from either of *x* or *y* to *z* passes through *w*.
- *x*, *y* ∈ *v*_*q* + 1_, …, *v*_*p*_. Therefore, MRCA (*x*, *y*) is *u*, and the shortest path from either of *x* or *y* to *z* passes through *u*.
- *x* ∈ *v*_1_, *v*_2_, …, *v*_*q*_ and *y* ∈ *v*_*q* + 1_, …, *v*_*p*_. Therefore, MRCA (*x*, *y*) is *u* and the shortest path from either of *x* or *y* to *z* passes through *u*.
- *y* ∈ *v*_1_, *v*_2_, …, *v*_*q*_ and *x* ∈ *v*_*q* + 1_, …, *v*_*p*_. Therefore, MRCA (*x*, *y*) is *u* and the shortest path from either of *x* or *y* to *z* passes through *u*.
- *x* ∈ *v*_1_, *v*_2_, …, *v*_*p*_ or *y* ∈ *v*_1_, *v*_2_, …, *v*_*p*_. Since *s*_*j*_≤_*i*_*s*_*k*_, therefore, there exists an MRCA for *x* and *y*. Let MRCA (*x*, *y*) be *q*. Therefore, the shortest path from either of *x* or *y* to *z* passes through *q*.
- *x*, *y* ∉ *v*_1_, *v*_2_, …, *v*_*p*_. Since, *s*_*j*_≤_*i*_*s*_*k*_, therefore, there exists and MRCA for *x* and *y* such that the shortest path from either of *x* or *y* to *z* passes through the MRCA (*x*, *y*).

Therefore, if the compatibility condition holds for *T*^′′^, and *T*′ is obtained using one refinement step from *T*^′′^, then the compatibility condition also holds for *T*′.

By induction, P is compatible with *T*′, as required.

Now we present a data structure that the algorithm uses to identify siblings. For the set of posets, P, a matrix *A* of size *n* × *n* is defined. We define

$$\begin{array}{ccc} A(i,j) & = & \left\{ \begin{array}{cc} \left|\{s_{x}|s_{j}{<}_{i}s_{x}\}\right| & \text{if}\;i\neq j;\\ -1 & \text{if}\;i=j. \end{array}\right. \end{array}$$

In other words, for *i* ≠ *j*, *A*(*i*, *j*) is the number of species *s*_*x*_ such that *s*_*j*_ is strictly less than *s*_*x*_ in the poset (*S*, ≤_*i*_).

**Theorem 2**. *Let* P *be a set of posets, and let A be the matrix representing* P. *If* P *is consistent, then A is symmetric*.

*Proof*. Let $P=\{P_{1},P_{2},\ldots ,P_{n}|P_{i}\;\text{is}\;\text{an}\;s_{i}-\text{poset}\}$ be a set of posets. P is *consistent* if, for all posets *P*_*i*_, *P*_*j*_ ∈ *P*, whenever *s*_*j*_ ≤_*i*_ *s*_*k*_, then *s*_*i*_ ≤_*j*_ *s*_*k*_. Let 1 ≤ *i* < *j* ≤ *n*. By the consistency condition, {*s*_*x*_∣*s*_*j*_<_*i*_*s*_*x*_} = {*s*_*x*_∣*s*_*i*_ < _*j*_*s*_*x*_}. Therefore, *A*(*i*, *j*) = *A*(*j*, *i*), and *A* is symmetric.

This *A* matrix represents an undirected graph, where siblings are indicated by cliques in the graph, that is, for a species *s*_*i*_, all other species connected to *s*_*i*_ with edges having equal labels, then they are siblings. Higher values indicate siblings at lower levels in the tree, in other words, the maximum value indicates leaf siblings. Note that if there is missing data or incorrect data in the posets, there will be a problem in constructing the tree, for example, if the posets have missing information or incorrect information then the algorithm will not be able to construct a tree for that specific gene corresponding to that posets set. To follow is an example to illustrate the defined data structures. Consider the set of posets P, where P is given as follows:

$$\begin{array}{ccc} P_{1} & = & \left(s_{1},s_{2}\right),\left(s_{1},s_{3}\right),\left(s_{1},s_{4}\right),\left(s_{2},s_{4}\right),\left(s_{3},s_{4}\right)\\ P_{2} & = & \left(s_{2},s_{1}\right),\left(s_{2},s_{3}\right),\left(s_{2},s_{4}\right),\left(s_{1},s_{4}\right),\left(s_{3},s_{4}\right)\\ P_{3} & = & \left(s_{3},s_{1}\right),\left(s_{3},s_{2}\right),\left(s_{3},s_{4}\right),\left(s_{1},s_{4}\right),\left(s_{2},s_{4}\right)\\ P_{4} & = & \left(s_{4},s_{1}\right),\left(s_{4},s_{2}\right),\left(s_{4},s_{3}\right) \end{array}$$

The matrix *A* corresponding to P is shown in Table 1

**Table 1:** Matrix *A* for the set of posets P.

	*s*_1_	*s*_2_	*s*_3_	*s*_4_
*s*_1_	-1	1	1	0
*s*_2_	1	-1	1	0
*s*_3_	1	1	-1	0
*s*_4_	0	0	0	-1

**Fig 9:**
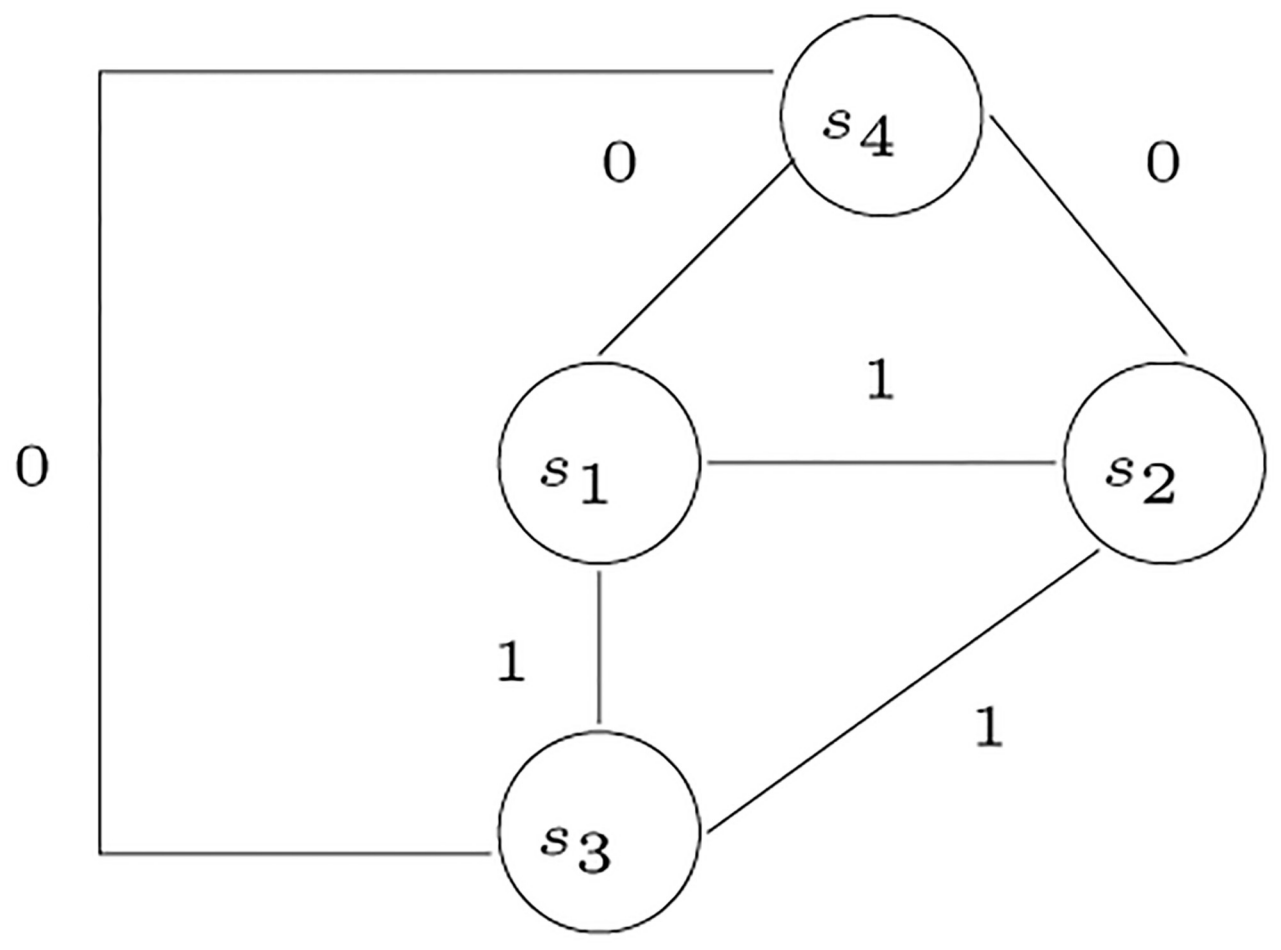
An undirected graph with cliques representing siblings.

And the graph *G* that is represented by the matrix *A* given in Table 1 is shown in Fig 9, where *s*_1_, *s*_2_, and *s*_3_ are siblings, and their parent and *s*_4_ are both children of the root.

**Fig 10:**
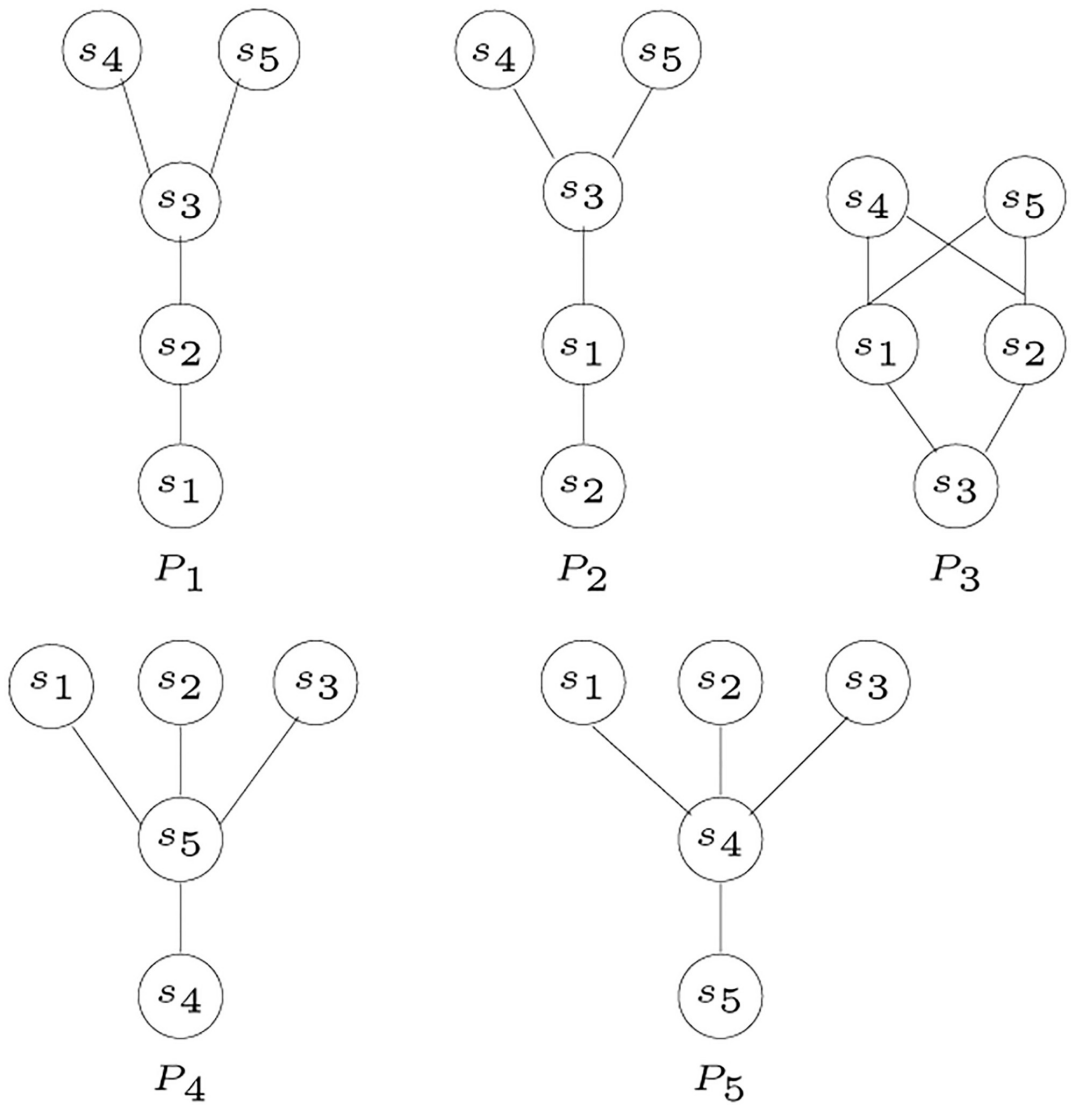
Diagram for posets [3].

To follow is an example to illustrate the data structures used in tree construction. The matrix shown in Table 2 is constructed for the posets in Fig 10.

**Table 2:** Matrix *A* for posets in Fig 10.

	*s*_1_	*s*_2_	*s*_3_	*s*_4_	*s*_5_
*s*_1_	-1	3	2	0	0
*s*_2_	3	-1	2	0	0
*s*_3_	2	2	-1	0	0
*s*_4_	0	0	0	-1	3
*s*_5_	0	0	0	3	-1

**Fig 11:**
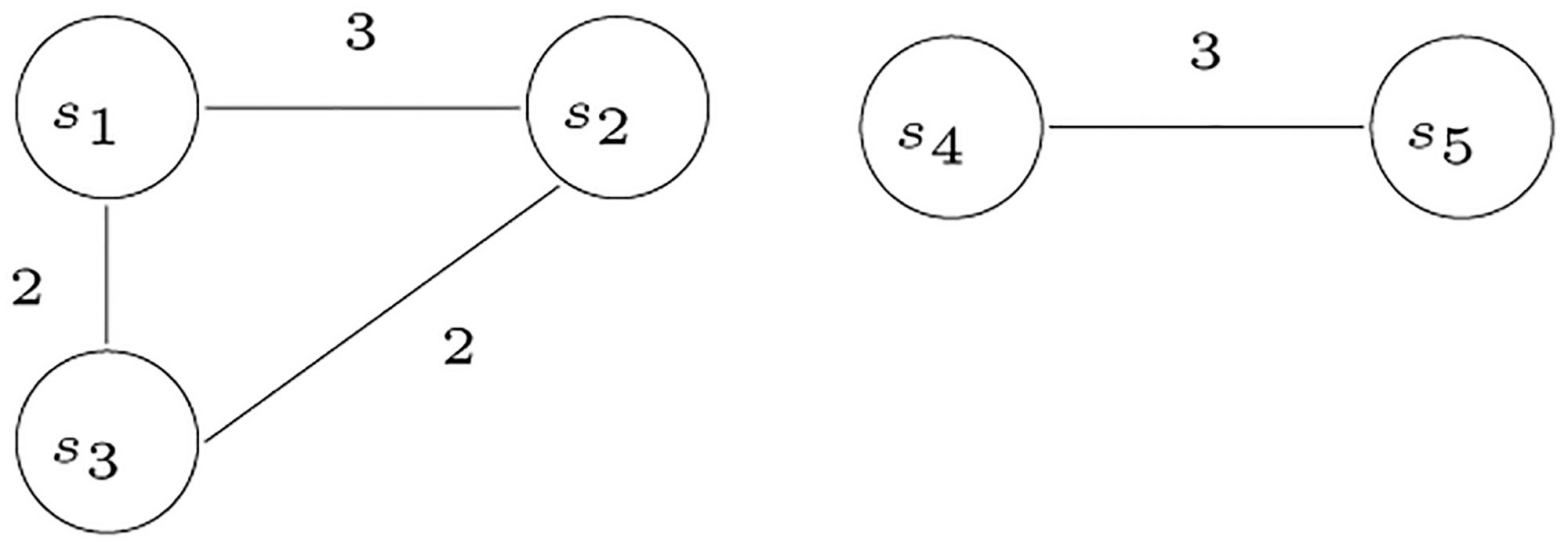
An undirected graph corresponding to the matrix shown in Table 2.

The graph in Fig 11 shows the cliques that represent siblings indicated by matrix *A* in Table 2.

The first row of matrix *A* indicates that *s*_2_ is a sibling of *s*_1_. The maximum value in the *s*_1_ row is 3, which is in the *s*_2_ column, and it is the only column with this value. This is also clear in the graph shown in Fig 11. Since the maximum value found in the *s*_1_ row is 3, and it is only under the *s*_2_ column, therefore, *s*_2_ is the only sibling of *s*_1_. Similarly, *s*_4_ and *s*_5_ are also siblings.

The algorithm starts by the procedure of inferring siblings by detecting cliques in the graph. For each species, the algorithm scans the row corresponding to that species, and detects which species are connected using edges with equal labels. The detected species are all siblings. After detecting each set of siblings comes the updating step. In this step, the rows and columns of the siblings are merged. This procedure is repeated until only one species is remaining, which is the root.

After scanning the *s*_1_ row, the matrix *A* is reduced as shown in Table 3.

**Table 3:** Matrix *A* for posets in Fig 10 after reducing *s*_1_ and *s*_2_.

	*x*	*s*_3_	*s*_4_	*s*_5_
*x*	-1	2	0	0
*s*_3_	2	-1	0	0
*s*_4_	0	0	-1	3
*s*_5_	0	0	3	-1

Similarly, the matrix *A* is reduced after detecting the siblings *s*_4_ and *s*_5_, as shown in Table 4.

**Table 4:** Updated matrix *A* for posets in Fig 10 after reducing *s*_4_ and *s*_5_.

	*x*	*s*_3_	*y*
*x*	-1	2	0
*s*_3_	2	-1	0
*y*	0	0	-1

This procedure is repeated, but this time the highest integer is 2, therefore, *s*_3_ is a sibling of *s*_12_, the parent of *s*_1_ and *s*_2_. And, the new matrix is shown in Table 5.

**Table 5:** Updated matrix *A* for posets in Fig 10.

	*z*	*y*
*z*	-1	0
*y*	0	-1

**Fig 12:**
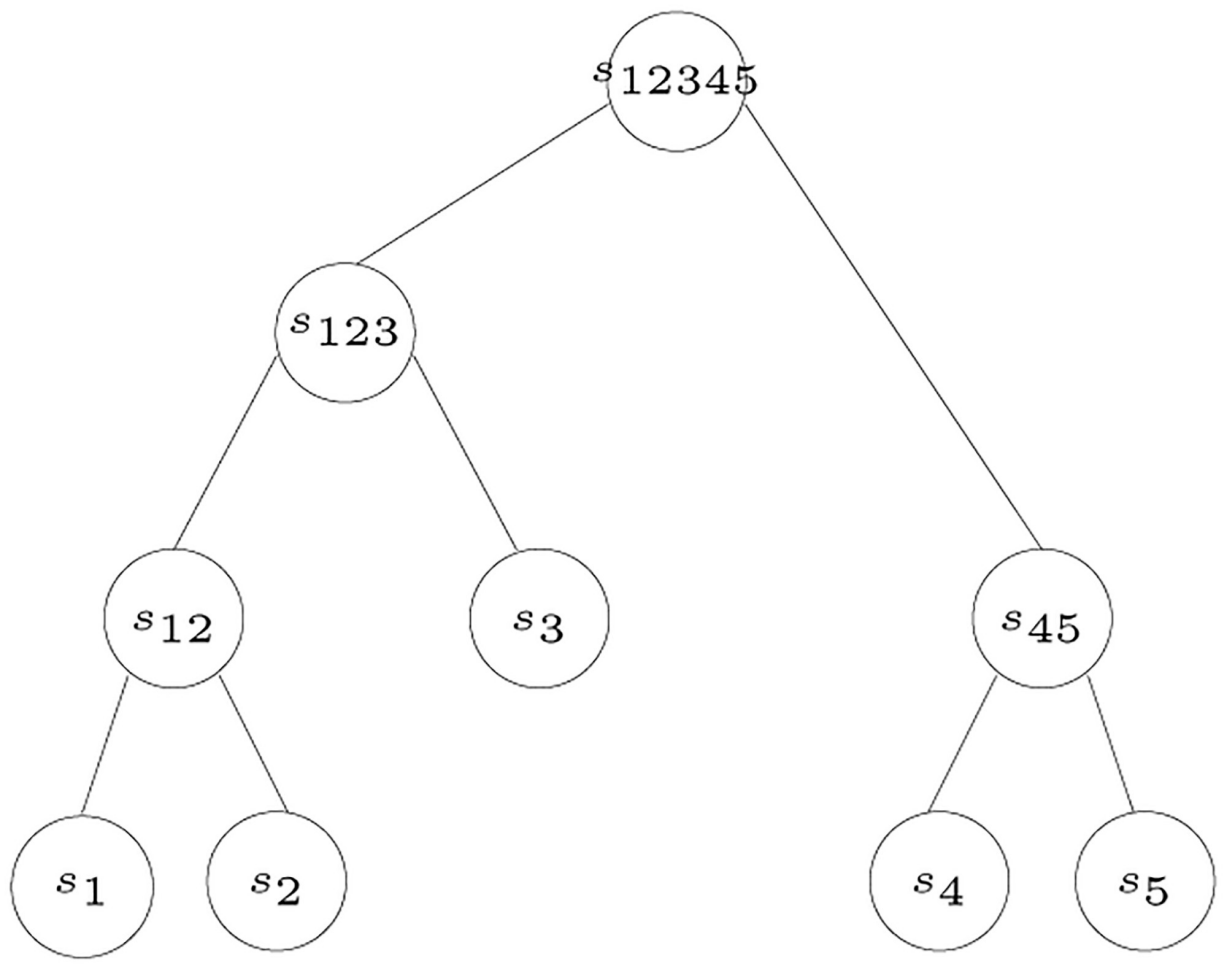
Tree corresponding to the posets in Fig 10.

The final step creates one root for the remaining species because all the values are 0, hence, all the remaining species are at the same level. The tree reconstructed from the posets in Fig 10 is shown in Fig 12.

**Fig 13:**
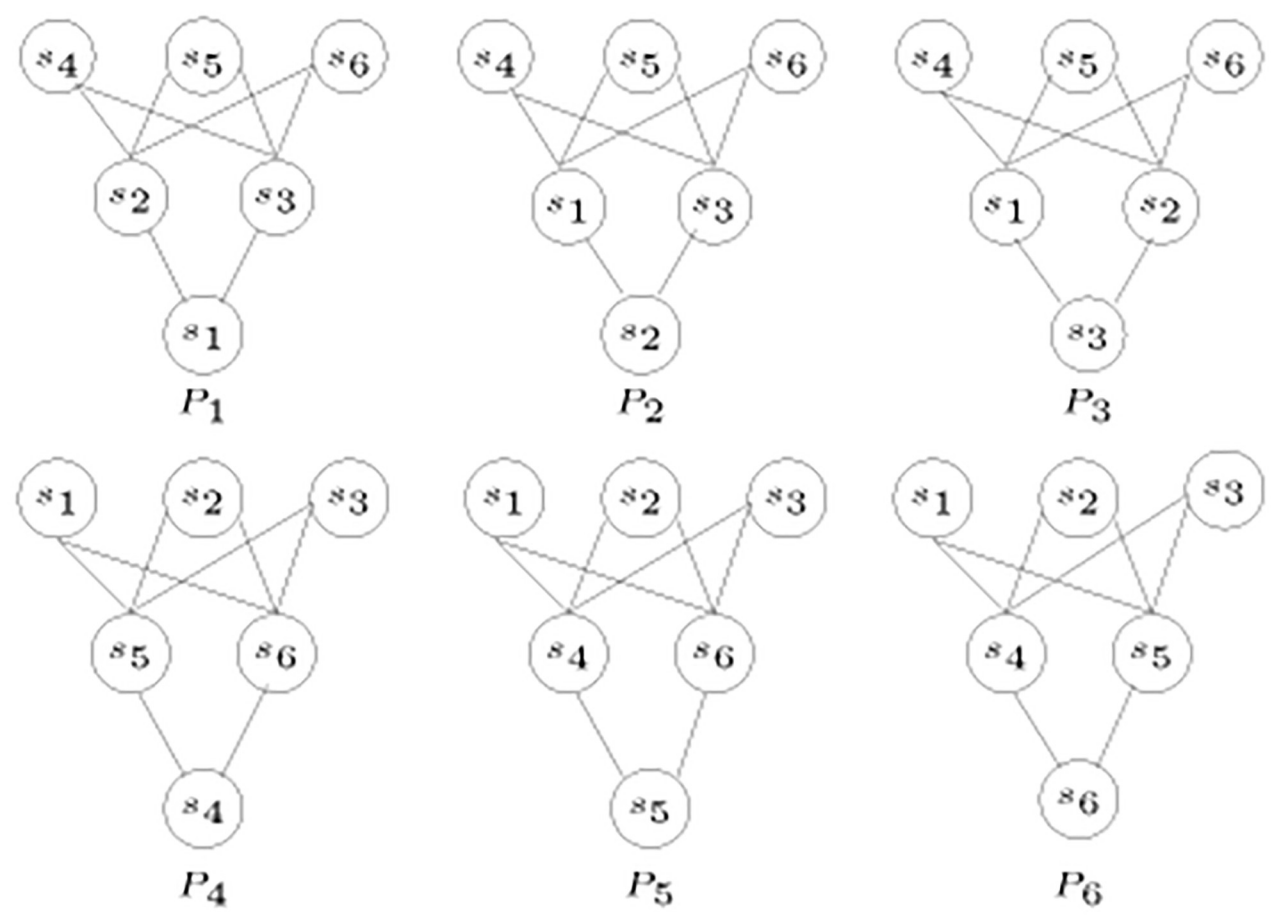
Diagrams for posets.

Another example to further illustrate the algorithm uses the set of posets P in Fig 13.

The matrix in Table 6 is constructed for the set of posets P in Fig 13.

**Table 6:** Matrix *A* for posets in Fig 13.

	*s*_1_	*s*_2_	*s*_3_	*s*_4_	*s*_5_	*s*_6_
*s*_1_	-1	3	3	0	0	0
*s*_2_	3	-1	3	0	0	0
*s*_3_	3	3	-1	0	0	0
*s*_4_	0	0	0	-1	3	3
*s*_5_	0	0	0	3	-1	3
*s*_6_	0	0	0	3	3	-1

The largest integer is 3, and it indicates that *s*_1_, *s*_2_, and *s*_3_ are siblings, as well as *s*_4_, *s*_5_, and *s*_6_.

The matrix then becomes as shown in Table 7.

**Table 7:** Updated matrix *A* for posets in Fig 13.

	*x*	*y*
*x*	-1	0
*y*	0	-1

**Fig 14:**
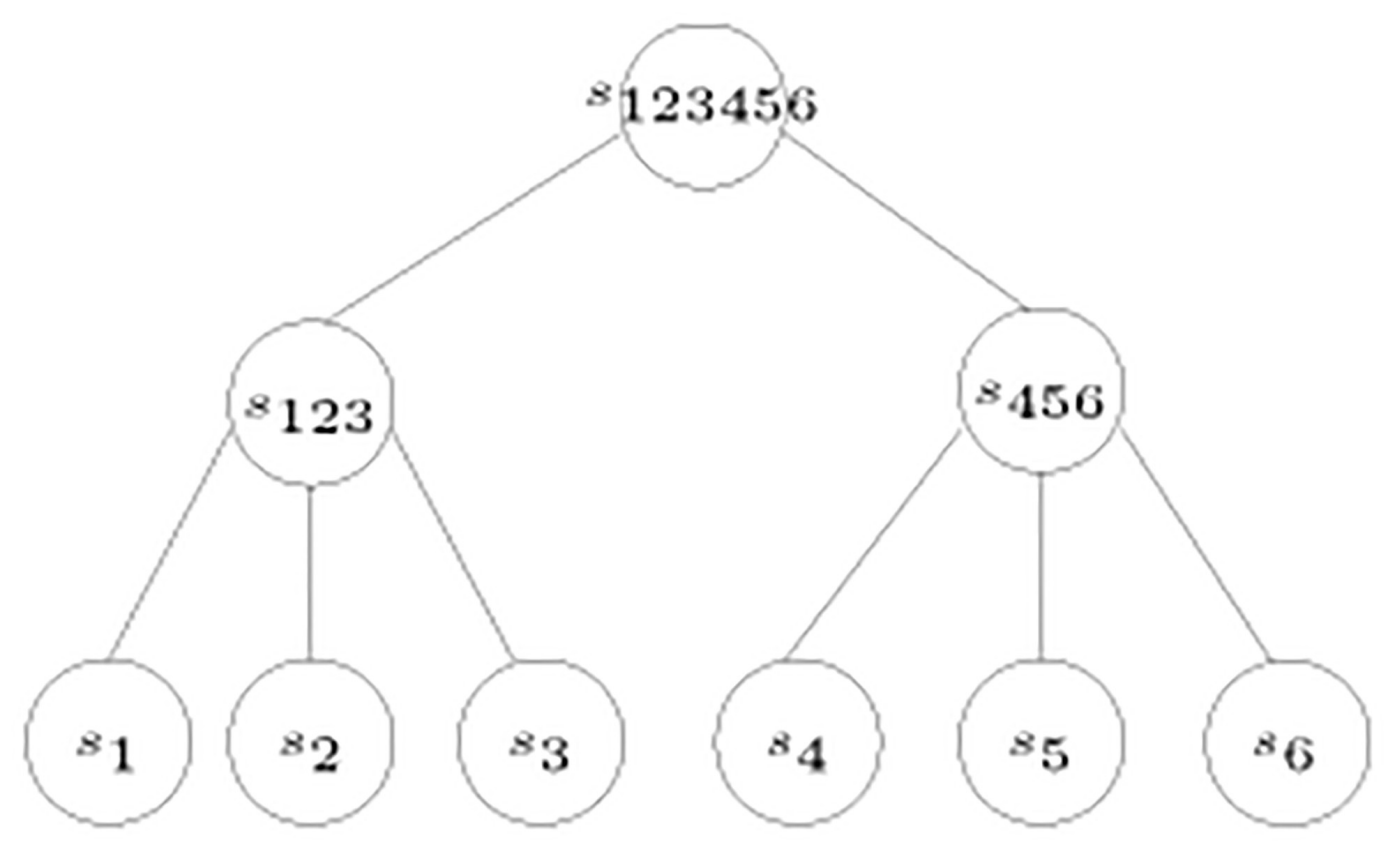
Tree corresponding to the posets in Fig 13.

Therefore, one root is created for the remaining two nodes to construct the tree in Fig 14.

To follow is an example to illustrate how the algorithm works to construct an *S*-tree from a set of posets $P=\{P_{1},P_{2},\ldots ,P_{n}\}$.

**Fig 15:**
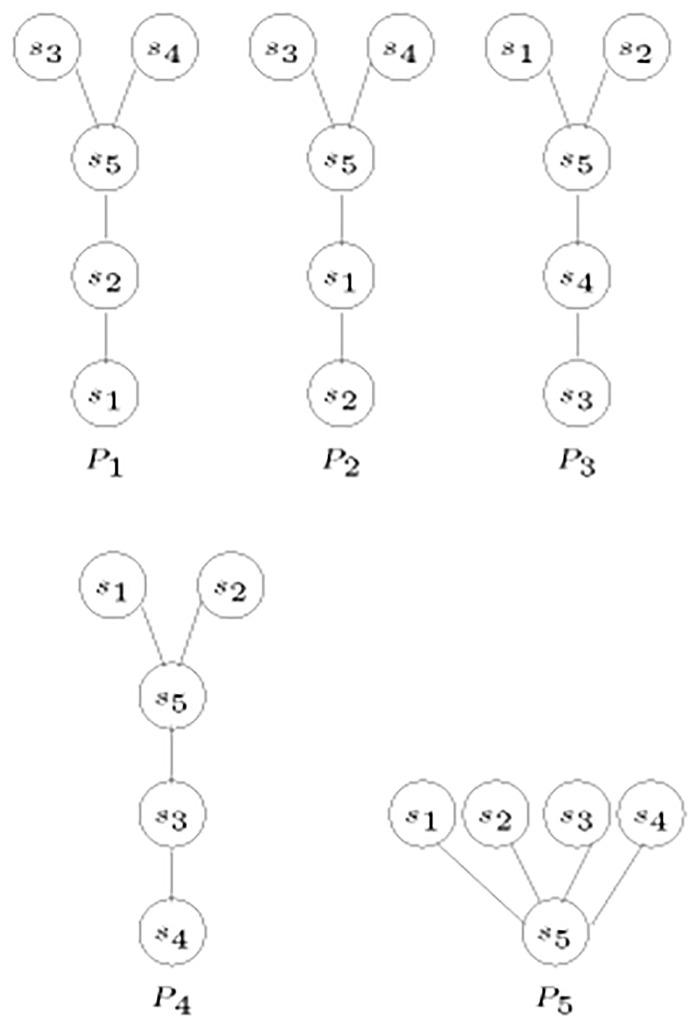
Set of posets P for the set of species *S* = {*s*_1_, *s*_2_, *s*_3_, *s*_4_, *s*_5_}.

Given a set of species, *S* = {*s*_1_, *s*_2_, *s*_3_, *s*_4_, *s*_5_}, with the set of posets P in Fig 15.

The corresponding *A* matrix is shown in Table 8.

**Table 8:** Matrix *A* for the posets in Fig 15.

	*s*_1_	*s*_2_	*s*_3_	*s*_4_	*s*_5_
*s*_1_	-1	3	0	0	2
*s*_2_	3	-1	0	0	2
*s*_3_	0	0	-1	3	2
*s*_4_	0	0	3	-1	2
*s*_5_	2	2	2	2	-1

Therefore, the maximum is 3, with the siblings *s*_1_ and *s*_2_, as well as *s*_3_ and *s*_4_.

And, the matrix *A* becomes as shown in Table 9.

**Table 9:** Updated matrix *A* for the posets in Fig 15.

	*s*_12_	*s*_34_	*s*_5_
*s*_12_	-1	0	2
*s*_34_	0	-1	2
*s*_5_	2	2	-1

**Fig 16:**
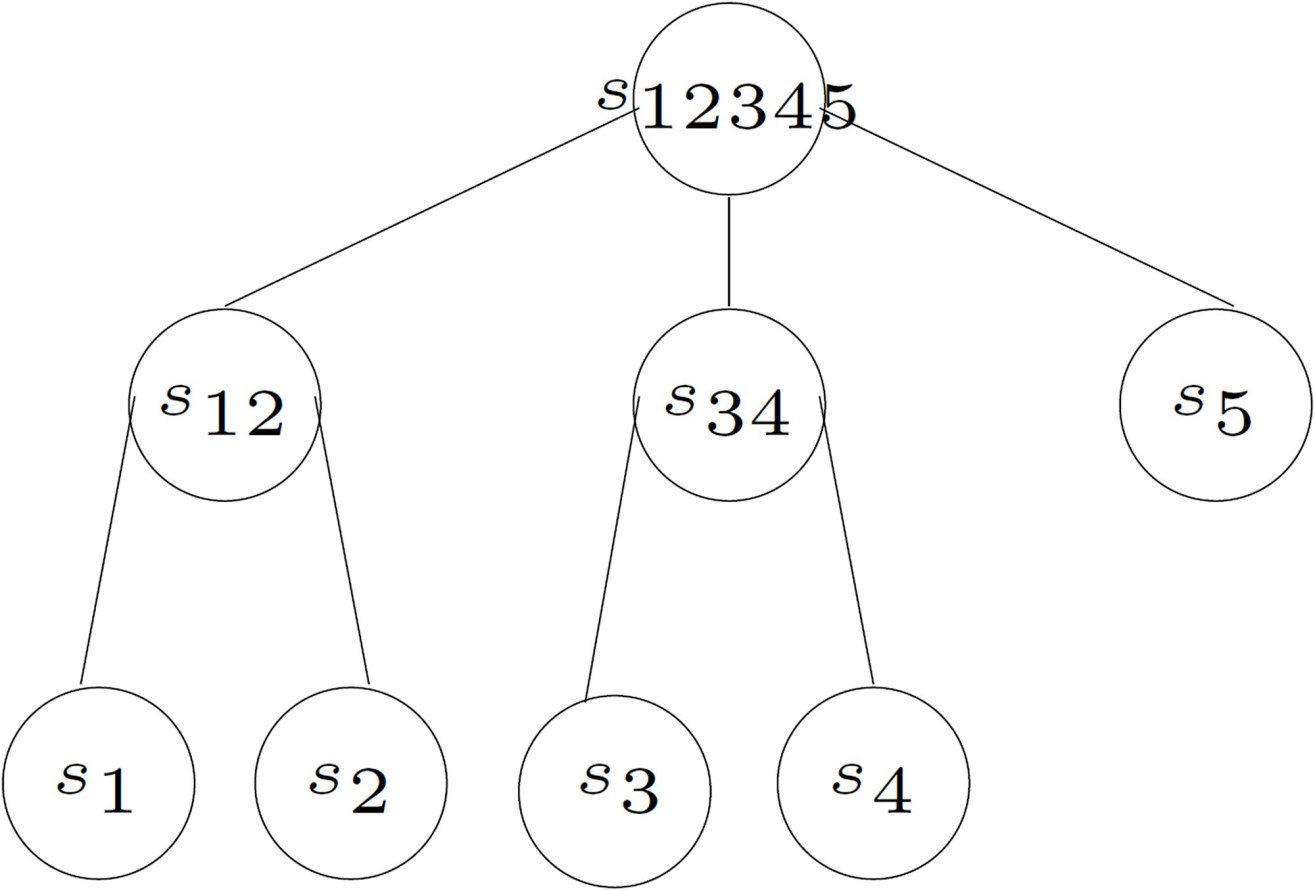
The tree corresponding to the set of posets P in Fig 15.

Now, *s*_5_ is a sibling of both *s*_12_ and *s*_34_, giving one root for the three nodes. The constructed tree is shown in Fig 16.

**Fig 17:**
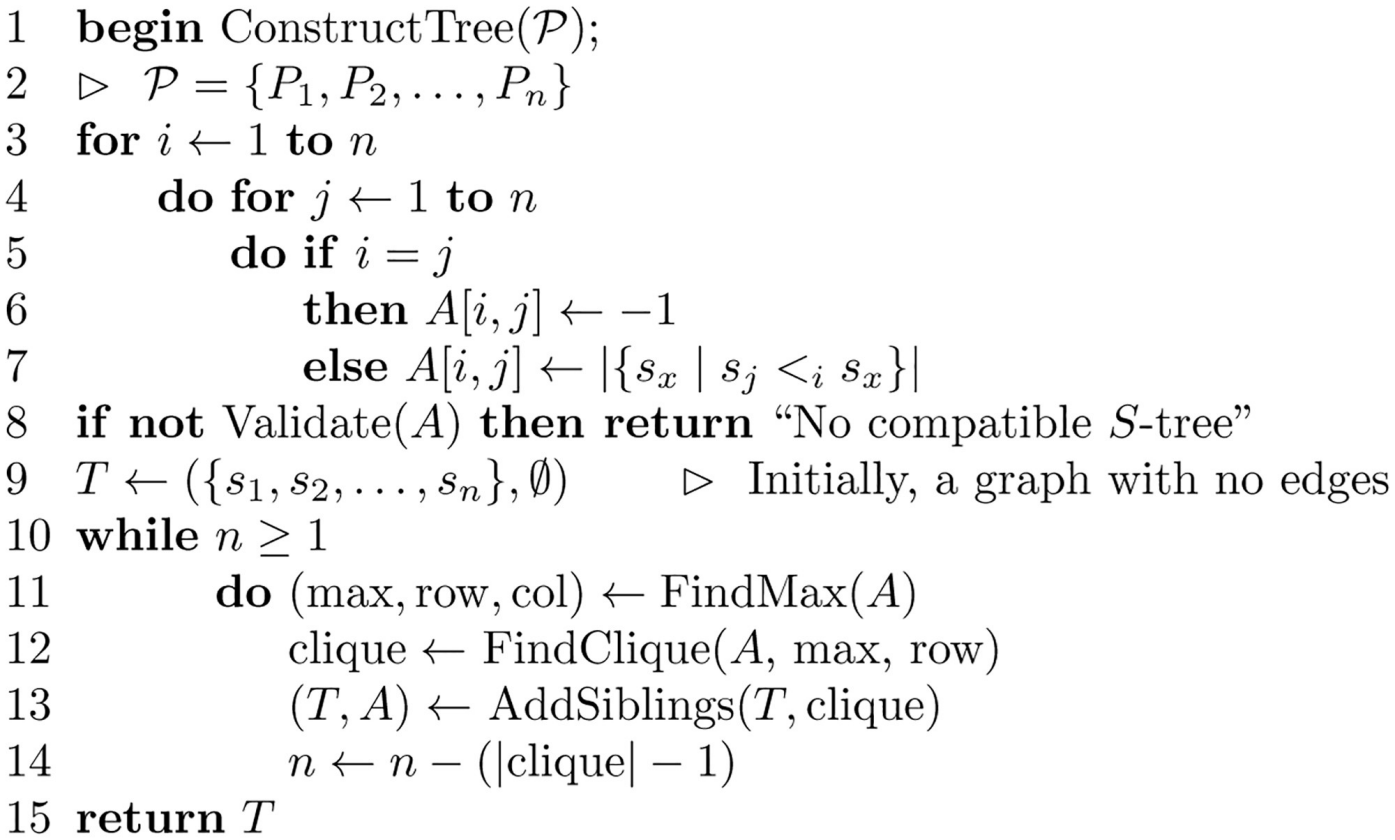
Algorithm to construct an *S*-tree from a set of posets $P=\{P_{1},P_{2},\ldots ,P_{n}\}$.

Fig 17 shows the algorithm for reconstructing a tree from a set of posets P. The algorithm validates the matrix *A* by testing that *A*[*i*, *j*] = *A*[*j*, *i*], for all *i* and *j*, where 1 ≤ *i* ≤ *n* and 1 ≤ *j* ≤ *n*. The algorithm also uses a subroutine to find cliques with equal edge labels. The subroutine scans the matrix *A* to find a clique with maximum edge labels. The subroutine AddSiblings shows the steps for adding the vertices that belong to a certain clique as siblings in the tree *T*. The subroutine also reduces the graph by merging the rows and columns in the matrix *A*.

**Fig 18:**
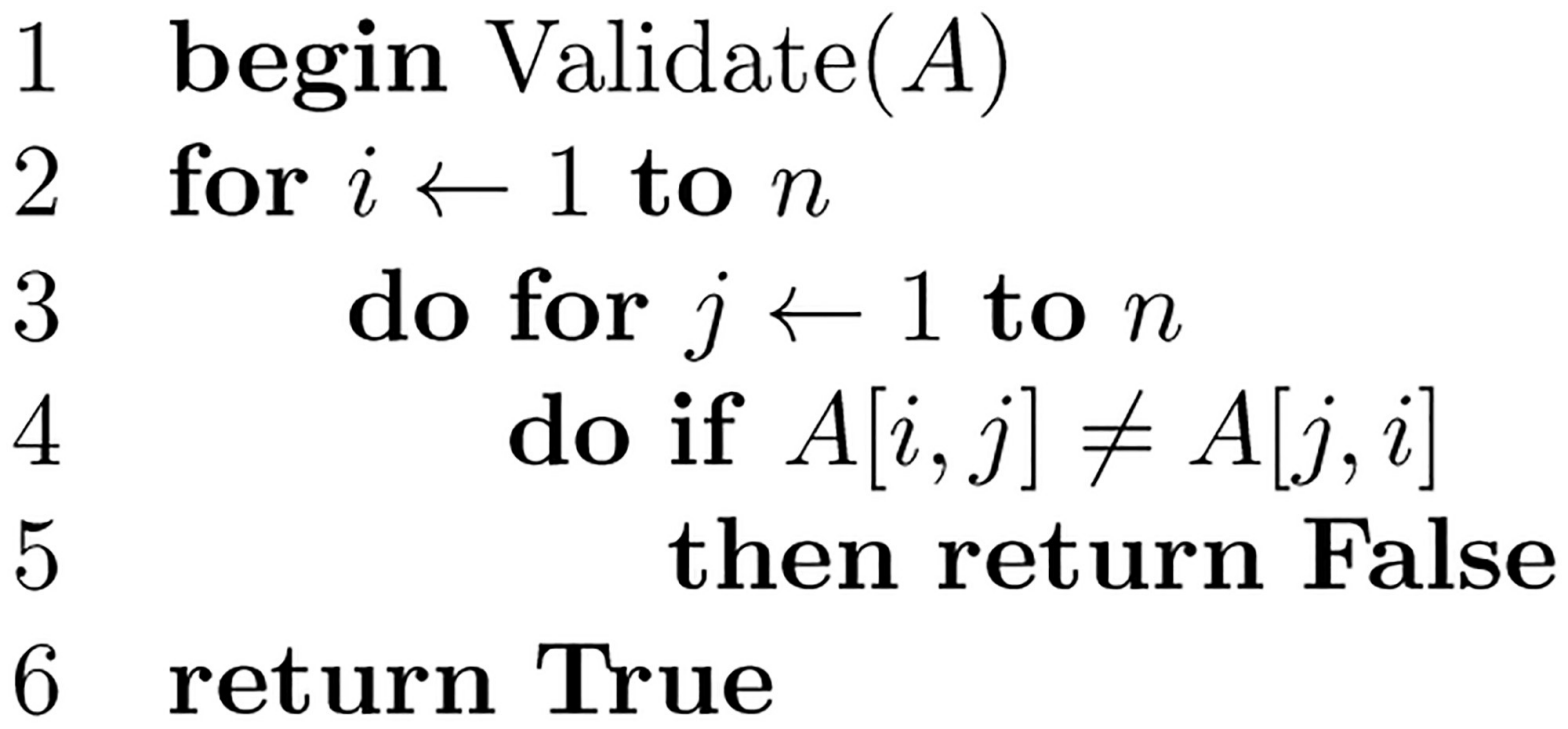
Algorithm to validate an *n* × *n* matrix *A*.

**Fig 19:**
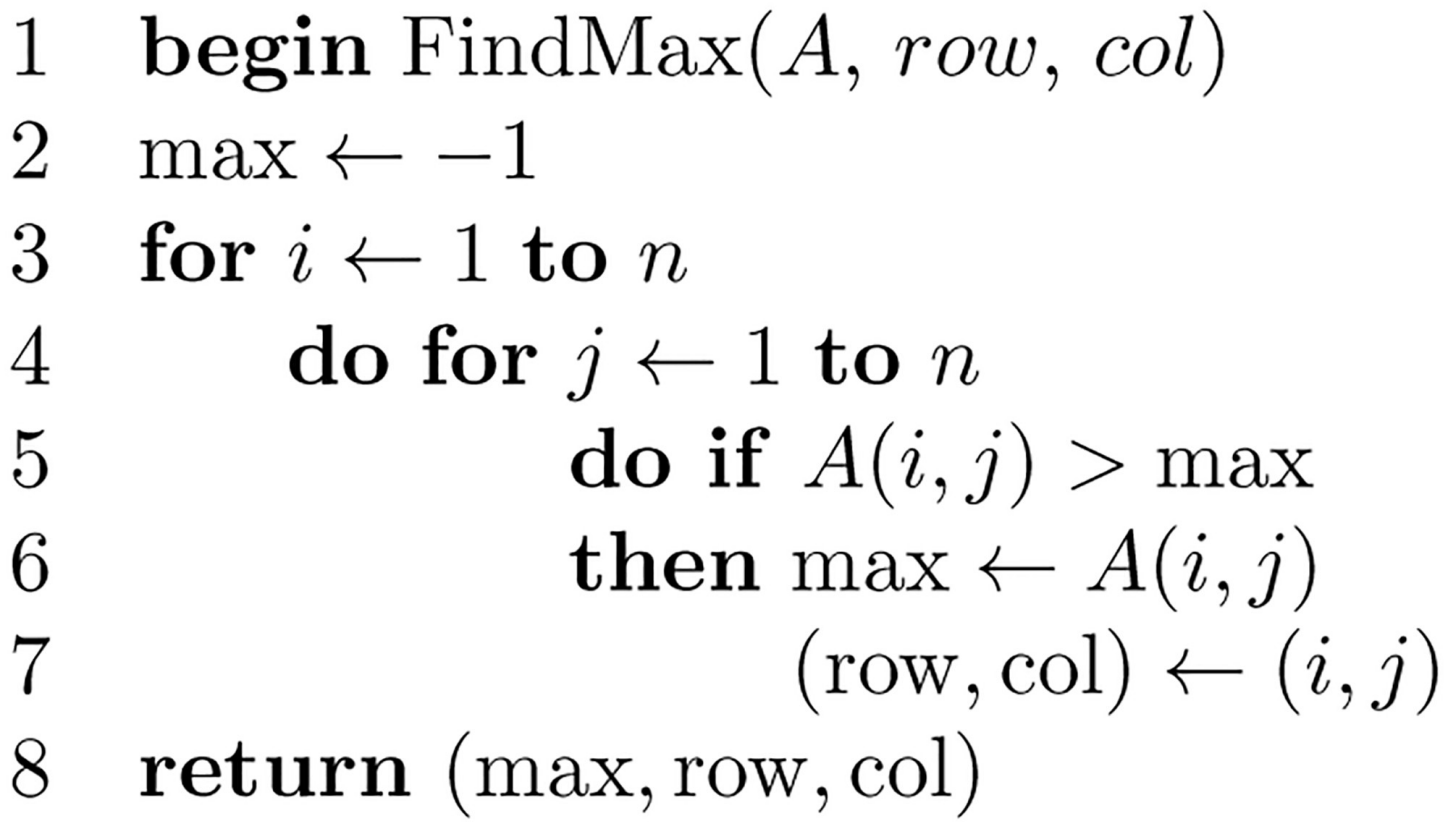
Algorithm to find the maximum of a matrix *A*.

**Fig 20:**
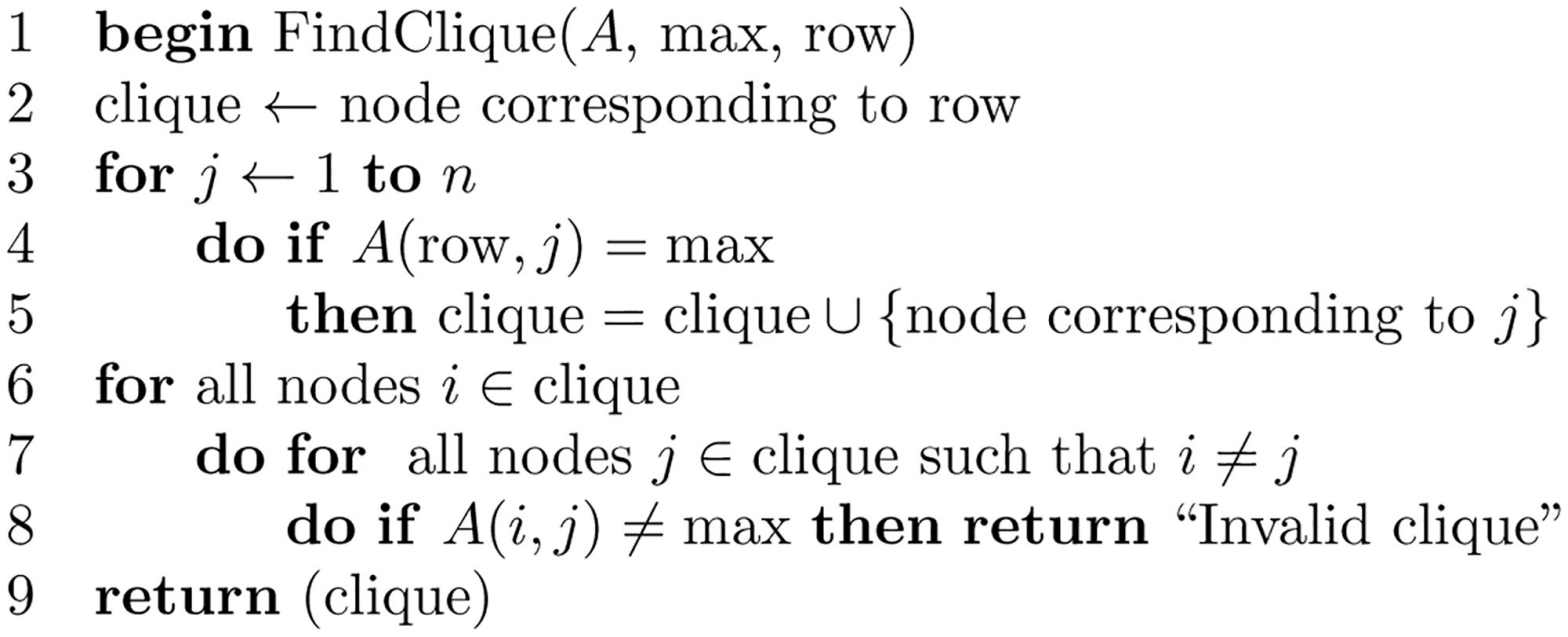
Algorithm to find a clique with edge labels equal *max*.

**Fig 21:**
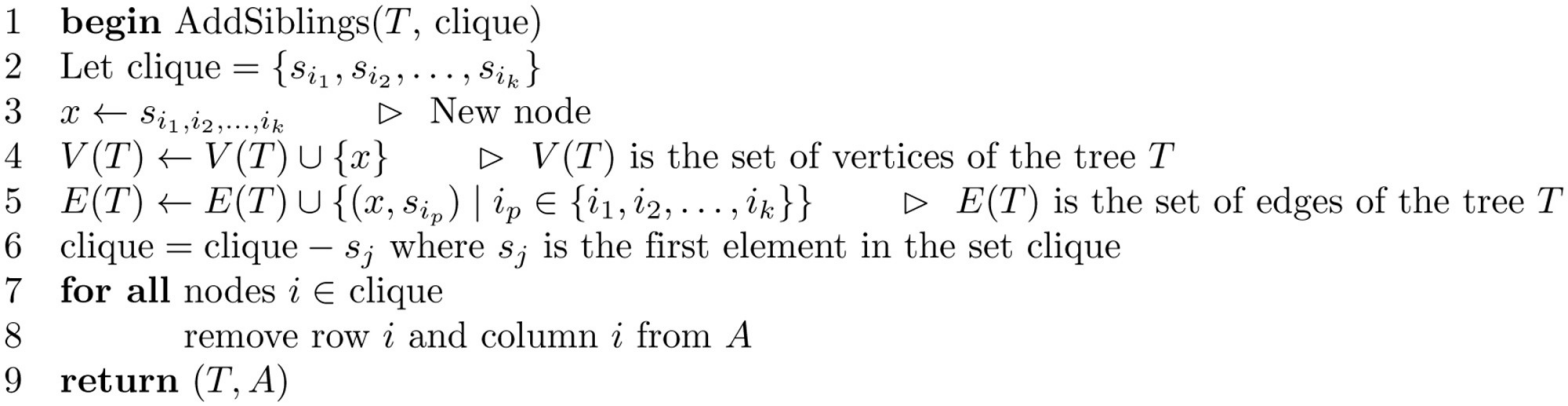
Algorithm to add elements of a clique as siblings in a tree *T*.

Fig 18 shows the subroutine for validating the matrix *A*. And, Fig 19 shows the subroutine that finds the maximum value stored in the matrix *A*, where Fig 20 is the subroutine that finds the clique with edge labels equal to the maximum value. The subroutine in Fig 21 adds the nodes in the clique found as siblings in the tree constructed.

**Theorem 3**. *The algorithm ConstructTree has O(n^3^) time complexity*.

*Proof*. Lines 2–6 in the algorithm ConstructTree contain two nested loops, each of which repeats *n* times. The statement in line 6, which is repeated in the nested loops, takes *O*(*n*) time, that is because the poset *P*_*i*_ contains, at most, *n* ordered pairs with *x* = *s*_*j*_. Therefore, the total amount for these three nested loops will be *O*(*n*^3^). Lines 8 scans the matrix *A* in *O*(*n*^2^). The while loop on line 10 repeats at most *n* times, on line 11, FindMax is *O*(*n*^2^), on line 12, FindClique is *O*(*n*^2^), AddSiblings on line 13 is *O*(*n*), Therefore, the while loop takes *O*(*n*^3^). Therefore, the complexity of the algorithm is *O*(*n*^3^).

**Theorem 4**. *The algorithm ConstructTree solves the* Compatible Tree Construction *problem*.

*Proof*. To prove the theorem, we use induction on the number of species. Let the number of species be *n*. For *n* = 1 and *n* = 2, there is no maximum value in the matrix *A*, hence, the tree is trivial. For *n* = 3, there are three possibilities for the third species *s*_3_. Either *s*_3_ is a sibling of *s*_1_ and *s*_2_, a sibling of their parent, or a sibling of either one of them. The algorithm checks the values in the *A* matrix, if *A*(1, 3) = *A*(2, 3) = *A*(1, 2), then *s*_3_ is a sibling of *s*_1_ and *s*_2_, otherwise, *s*_3_ is a sibling of their parent. In case of *s*_1_ and *s*_2_ not being siblings, then the values in the *A* matrix will detect *s*_3_ as a sibling of either one of them, that is the third possibility. After detecting siblings, the matrix *A* is reduced by eliminating the siblings and replacing them by their parent. Therefore, for *n* species, the algorithm scans the matrix *A*, and at each step, the siblings are eliminated and replaced by their parent, this reduces the matrix *A*, until only one species is remaining, which is the root.

## Generating a set of posets from a given *S*-tree

For each tree *T*, there exists a set of posets P compatible with *T*. In this section, we show how given a tree *T*, the set of compatible posets can be generated.

A set of posets P is compatible with an *S*-tree *T* if, for all distinct triples *x*, *y*, *z* ∈ *L*(*T*) such that *λ*(*x*) = *s*_*i*_, *λ*(*y*) = *s*_*j*_, and *λ*(*z*) = *s*_*k*_ and such that *s*_*j*_≤_*i*_*s*_*k*_, then we have the shortest path from either of *x* or *y* to *z* passes through MRCA (*x*, *y*). Therefore, the procedure of obtaining posets from a tree is straightforward. Given a tree *T*, it is clear which species are closer to each other than others, and hence, posets can be generated. By obtaining the path from each species (leaf node) to the root of the tree, and laying this path horizontally, we get the nodes sorted in order of closeness to this specific leaf node. Each node on the path represents a subtree, of which the leaves belonging to the species set represent one level of the poset.

**Fig 22:**
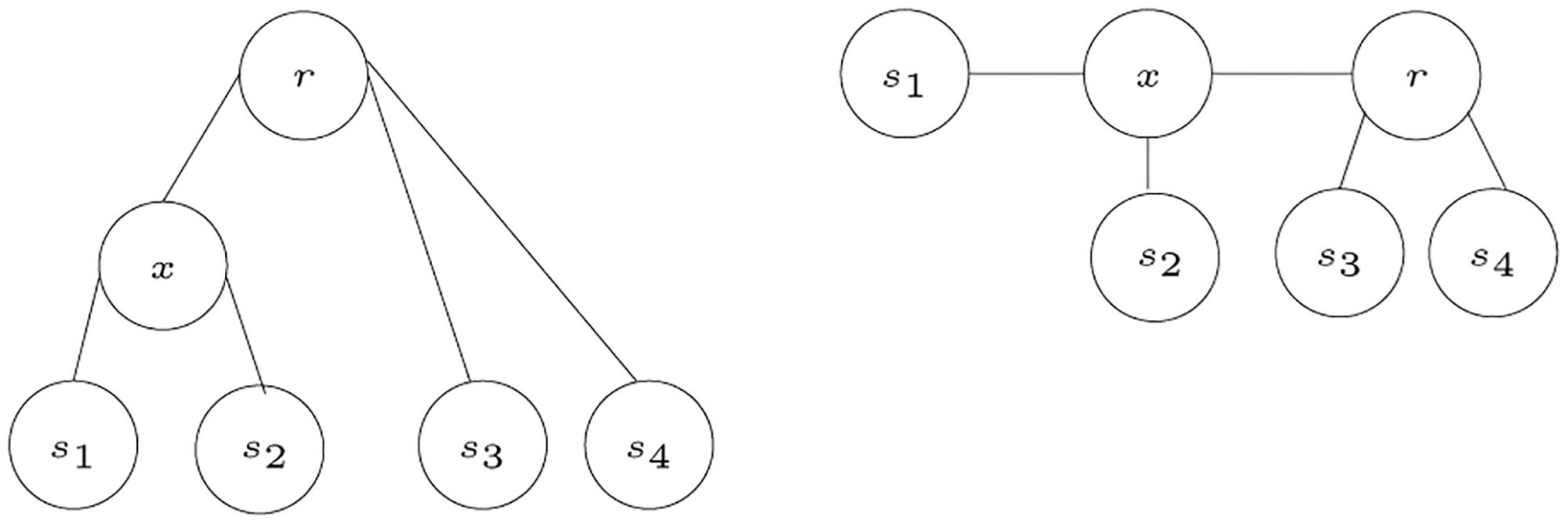
An example of how the poset corresponding to *s*_1_ is generated.

An example to illustrate how posets are generated from a tree is shown in Fig 22. The tree on the right shows the path from *s*_1_ to the root, where each node on the path is a root to a subtree, and the leaves belonging to each subtree represent a level of the poset *P*_1_. The subtree with the root *s*_1_ has only one leaf and that is *s*_1_. The second level of the poset contains the leaves in the subtree with the root *x*, and that is only *s*_2_, then comes the last level, in the subtree with the root *r*, and this subtree contains the leaves *s*_3_ and *s*_4_. Therefore, the poset *P*_1_ is generated as follows. *P*_1_ = {(*s*_1_, *s*_2_), (*s*_1_, *s*_3_), (*s*_1_, *s*_4_), (*s*_2_, *s*_3_), (*s*_2_, *s*_4_)}.

For example, given the tree shown in Fig 12, we look at each species to generate the corresponding poset. Starting with *s*_1_, the poset *P*_1_ automatically contains the ordered pairs (*s*_1_, *s*_2_), (*s*_1_, *s*_3_), (*s*_1_, *s*_4_), and (*s*_1_, *s*_5_). It is clear from the tree that *s*_2_ is the closest sibling to *s*_1_, this adds the ordered pairs (*s*_2_, *s*_3_), (*s*_2_, *s*_4_), and (*s*_2_, *s*_5_) to the poset *P*_1_. Also, the ordered pairs (*s*_3_, *s*_4_) and (*s*_3_, *s*_5_) are added. In a similar manner the posets *P*_2_, *P*_3_, *P*_4_, and *P*_5_ are generated as shown in Fig 10.

**Fig 23:**
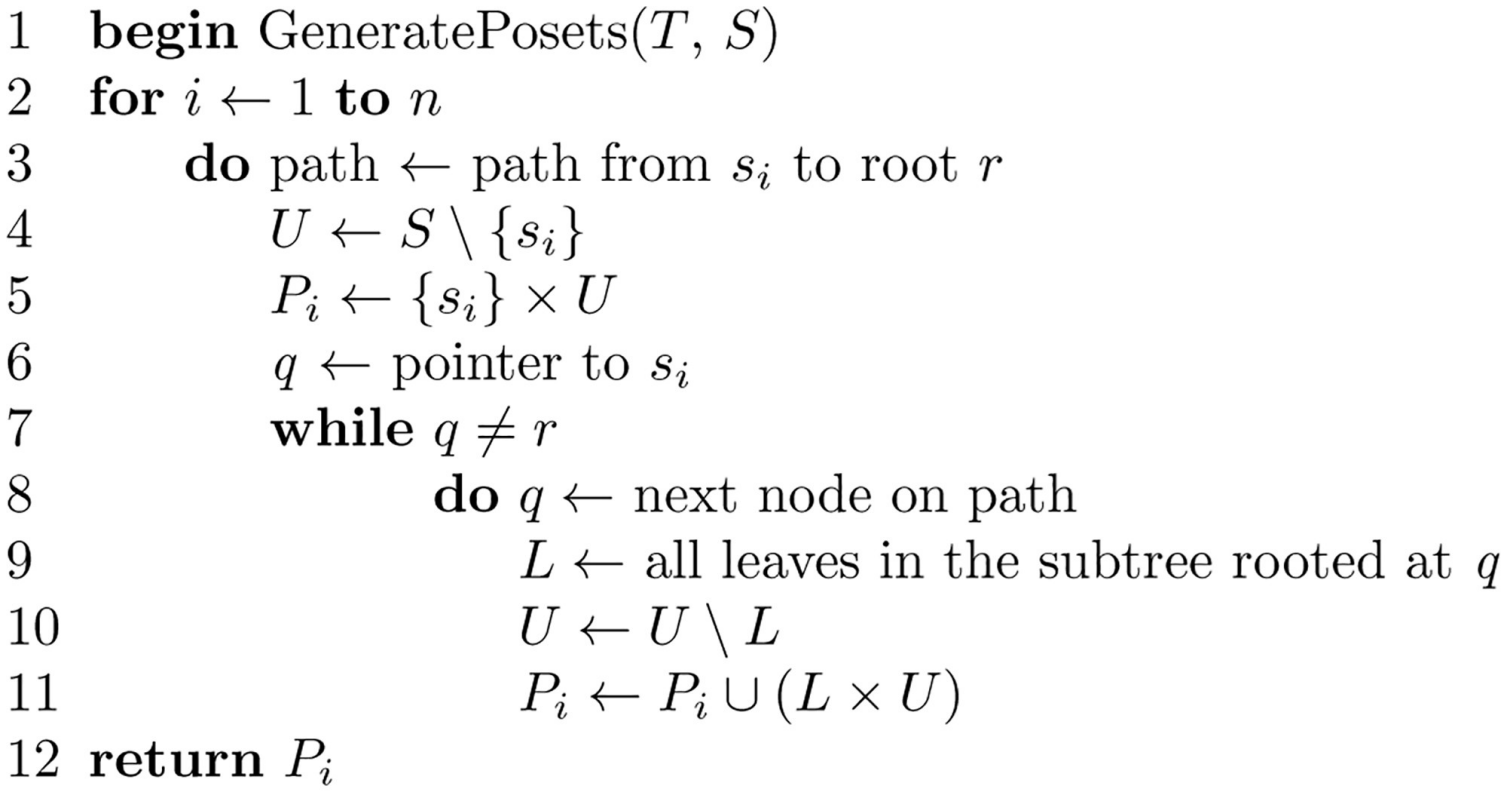
Algorithm to generate a set of posets P from an *S*-tree *T*.

**Theorem 5**. *The algorithm GeneratePosets shown in* Fig 23 *generates the set of posets* P *that is compatible with a given tree T*.

*Proof*. Using a proof by construction, we show that the algorithm GeneratePosets generates the set of posets P compatible with a given tree *T*. From the definition of compatible in Section 2 of the main document, we know that an *s*_*i*_-poset *P*_*i*_ = (*S*, ≤_*i*_) is compatible with *S*-tree *T* if, for all distinct triples *x*, *y*, *z* ∈ *L*(*T*) such that *λ*(*x*) = *s*_*i*_, *λ*(*y*) = *s*_*j*_, and *λ*(*z*) = *s*_*k*_ and such that *s*_*j*_≤_*i*_*s*_*k*_, then we have the shortest path from either of *x* or *y* to *z* passes through MRCA (*x*, *y*). The algorithm GeneratePosets finds, for a species *s*_*i*_, the path *p* from *s*_*i*_ to the root *r*, on that path, the nodes that come first on the path *p* are definitely closer to *s*_*i*_ and, hence, come at a lower level in the poset. That follows from the definition of compatible, which indicates that if *s*_*j*_≤_*i*_*s*_*k*_, then the shortest path from either of *x* or *y* to *z* passes through MRCA (*x*, *y*). Therefore, by scanning the path *p*, the set of posets P can be constructed.

**Theorem 6**. *The algorithm GeneratePosets has a time complexity of* *O*(*n*^3^).

*Proof*. Let the number of species be *n*. The loop on line 2 iterates *n* times, and on line 3, finding the path from a certain species to the root is also linear in the number of species, this gives a complexity *O*(*n*^2^). Then on line 7, the while loop is also linear in *n*, and on line 9, finding all leaves in a subtree is linear as well. This gives a total complexity of *O*(*n*^3^).

## Relating posets to trees

The following theorems relate posets and trees to one another.

**Theorem 7**. *Given a set of posets* P, *if there exists an S-tree T that* P *is compatible with, then T can be used to generate the same set of posets* P.

*Proof*. Given a set of posets P, assume that P is compatible with a tree *T*. Assume that *T*, in turn, generates a different set of posets $P^{\prime }$. $P^{\prime }$ can now be used to construct a tree *T*′ that is compatible with $P^{\prime }$, *T*′ is expected to be equivalent to *T*. However, since $P^{\prime }$ and P are not equal, then the two trees constructed are also not the same. Since, *T* and *T*′ are different, therefore, *T* and *T*′ can yield contradictory 2-partitions, this means that that *T* and *T*′ may be contradictory trees, and hence, one of them can not be used to give the same set of posets. Hence, there is a contradiction, and *T* can not be used to generate a set of posets other than P.

**Theorem 8**. *Let* $P_{1}$ *and* $P_{2}$ *be two sets of posets that are compatible with the two S-trees, T_1_ and T_2_*. *Then T_1_ and T_2_ are contradictory if and only if there exists a poset* $P_{i}\in P_{1}$ *and* $P_{j}\in P_{2}$, *such that* $P_{i}\in P_{1}$ *is inconsistent with* $P_{j}\in P_{2}$.

*Proof*. First, we prove that if *T*_1_ and *T*_2_ are contradictory then there exists a poset $P_{i}\in P_{1}$ and a poset $P_{j}\in P_{2}$, such that $P_{i}\in P_{1}$ is inconsistent with $P_{j}\in P_{2}$. Using a proof by contradiction, assume that *T*_1_ and *T*_2_ are contradictory and there is no poset $P_{i}\in P_{1}$ and $P_{j}\in P_{2}$, such that $P_{i}\in P_{1}$ is inconsistent with $P_{j}\in P_{2}$. Since, *T*_1_ and *T*_2_ are contradictory, therefore, there exists an edge in *T*_1_ and an edge in *T*_2_, that when cut induces contradictory 2-partitions. This means that there exists four species *s*_1_, *s*_2_, *s*_3_, and *s*_4_, such that *s*_1_ and *s*_2_ belong to the same partition in one tree but not in the other. Similarly, *s*_3_ and *s*_4_ belong to the same partition in one tree but not in the other. Since, the set of posets $P_{1}$ is compatible with *T*_1_ and the set of posets $P_{2}$ is compatible with *T*_2_, and since *T*_1_ and *T*_2_ are contradictory, therefore, there exists a poset $P_{i}\in P_{1}$ and a poset $P_{j}\in P_{2}$ such that $P_{i}\in P_{1}$ is inconsistent with $P_{j}\in P_{2}$. This leads to a contradiction with the assumption.

The second part of the proof proves that if there exists a poset $P_{i}\in P_{1}$ and a poset $P_{j}\in P_{2}$, such that $P_{i}\in P_{1}$ is inconsistent with $P_{j}\in P_{2}$, then *T*_1_ and *T*_2_ are contradictory. Using a proof by contradiction, assume that there exists a poset $P_{i}\in P_{1}$ and a poset $P_{j}\in P_{2}$, such that $P_{i}\in P_{1}$ is inconsistent with $P_{j}\in P_{2}$ while *T*_1_ and *T*_2_ are non-contradictory. If $P_{i}\in P_{1}$ is inconsistent with $P_{j}\in P_{2}$, therefore, $P_{1}$ is inconsistent with the set of posets $P_{2}$, hence, the two sets of posets can create contradictory 2-partitions in their corresponding trees, and therefore, the trees that are compatible with both sets of posets can not be non-contradictory, and this leads to a contradiction with the assumption. Therefore, the theorem follows.

**Fig 24:**
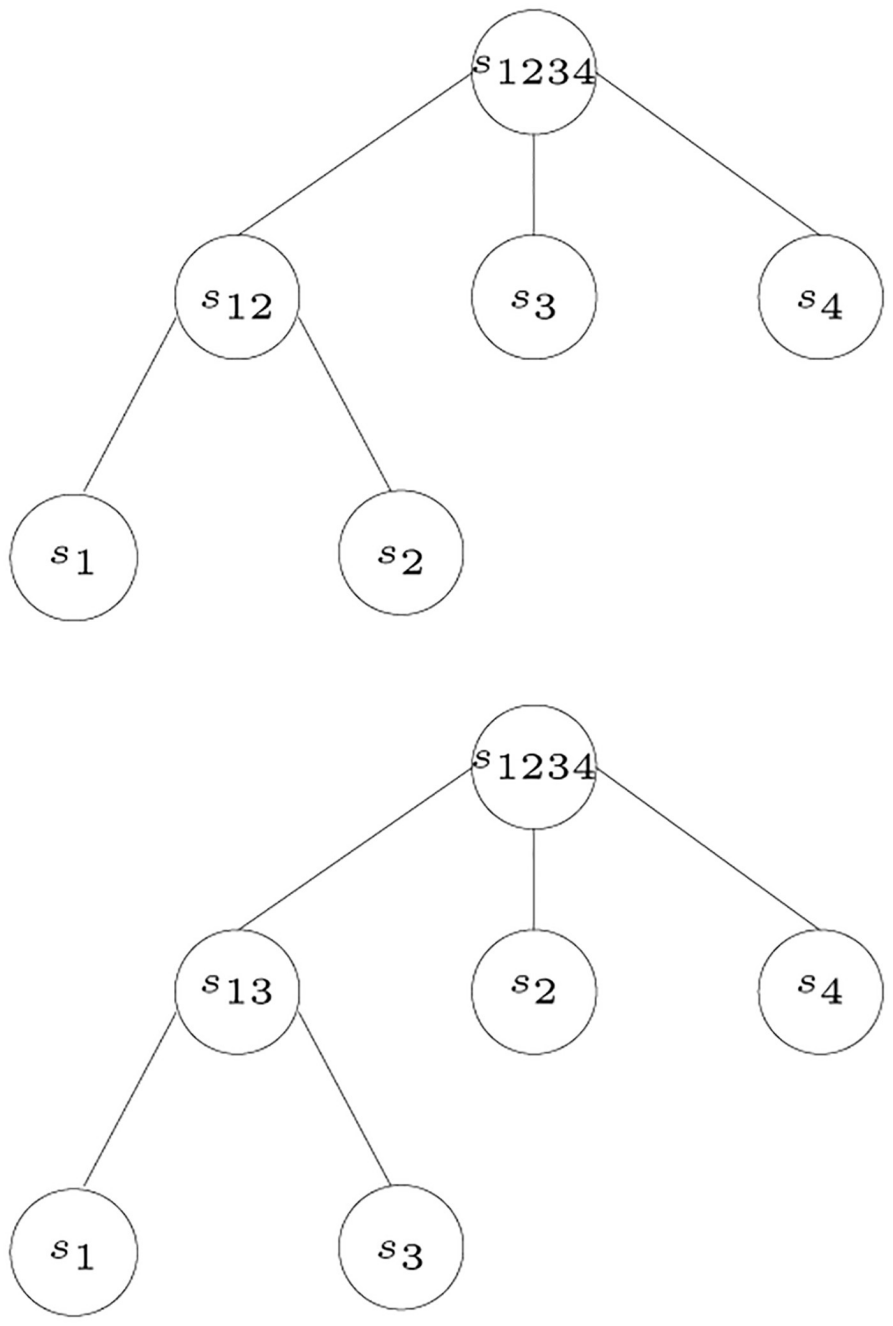
Two contradicting trees.

Fig 24 shows an example to illustrate Theorem 8. The set of posets $P_{1}$ corresponding to the tree at the top consists of the following posets:

$$\begin{array}{ccc} P_{1} & = & \left(s_{1},s_{2}\right),\left(s_{1},s_{3}\right),\left(s_{1},s_{4}\right),\left(s_{2},s_{3}\right),\left(s_{2},s_{4}\right)\\ P_{2} & = & \left(s_{2},s_{1}\right),\left(s_{2},s_{3}\right),\left(s_{2},s_{4}\right),\left(s_{1},s_{3}\right),\left(s_{1},s_{4}\right)\\ P_{3} & = & \left(s_{3},s_{1}\right),\left(s_{3},s_{2}\right),\left(s_{3},s_{4}\right)\\ P_{4} & = & \left(s_{4},s_{1}\right),\left(s_{4},s_{2}\right),\left(s_{4},s_{3}\right) \end{array}$$

And, the set of posets $P_{2}$ corresponding to the tree at the bottom consists of the following posets:

$$\begin{array}{ccc} P_{1} & = & \left(s_{1},s_{2}\right),\left(s_{1},s_{3}\right),\left(s_{1},s_{4}\right),\left(s_{3},s_{2}\right),\left(s_{3},s_{4}\right)\\ P_{2} & = & \left(s_{2},s_{1}\right),\left(s_{2},s_{3}\right),\left(s_{2},s_{4}\right)\\ P_{3} & = & \left(s_{3},s_{1}\right),\left(s_{3},s_{2}\right),\left(s_{3},s_{4}\right),\left(s_{1},s_{3}\right),\left(s_{1},s_{4}\right)\\ P_{4} & = & \left(s_{4},s_{1}\right),\left(s_{4},s_{2}\right),\left(s_{4},s_{3}\right) \end{array}$$

The poset $P_{1}\in P_{1}$ indicates that *s*_2_ is a sibling of *s*_1_, while the poset $P_{1}\in P_{2}$ indicates that *s*_3_ is a sibling of *s*_1_. Therefore, the two posets are inconsistent.

## Refinement of trees

We start with a basic result about refinement (Theorem 9).

**Lemma 1**. *Let T be an S-tree. Let Q be the 2-partition set of T. Then Q is not contradictory with itself*.

*Proof*. We show that every pair of 2-partitions in *Q* is non-contradictory. Consider an arbitrary pair of distinct edges of *T*. This pair of edges are the ends of a unique path in *T*. Let *u*_0_, *u*_1_, …, *u*_*k* − 1_, *u*_*k*_ be that path. Then the edges are (*u*_0_, *u*_1_) and (*u*_*k* − 1_, *u*_*k*_). These edges partition *S* into three sets: *X*, the set of species reachable from *u*_0_ without using (*u*_0_, *u*_1_); *Y*, the set of species reachable from *u*_*k*_ without using (*u*_*k* − 1_, *u*_*k*_); and *Z*, the set of species reachable from *u*_1_, *u*_2_, …, *u*_*k* − 1_ without using (*u*_0_, *u*_1_) or (*u*_*k* − 1_, *u*_*k*_). The 2-partition corresponding to (*u*_0_, *u*_1_) is (*X*, *Y* ∪ *Z*), and the 2-partition corresponding to (*u*_*k* − 1_, *u*_*k*_) is (*X* ∪ *Z*, *Y*). Recall the definition of contradictory 2-partitions: Two 2-partitions *X* = (*X*_1_, *X*_2_) and *Y* = (*Y*_1_, *Y*_2_) are contradictory partitions if there exist four species *s*_1_, *s*_2_, *s*_3_, *s*_4_ such that *s*_1_, *s*_2_ ∈ *X*_1_, *s*_3_, *s*_4_ ∈ *X*_2_, *s*_1_, *s*_3_ ∈ *Y*_1_, and *s*_2_, *s*_4_ ∈ *Y*_2_. Let *s*_1_, *s*_2_, *s*_3_, *s*_4_ ∈ *S*. If *s*_1_, *s*_2_ ∈ *X* and *s*_3_, *s*_4_ ∈ *Y* ∪ *Z*, then *s*_1_, *s*_2_ ∈ *X* ∪ *Z*, so the definition definitely does not apply to the 2-partitions corresponding to (*u*_0_, *u*_1_) and (*u*_*k* − 1_, *u*_*k*_). Since the two edges were arbitrary, we conclude that *Q* is not contradictory with itself.

**Lemma 2**. *Let T*_1_ *be an S-tree, and let T*_2_ *be a refinement of T*_1_. *Let Q*_1_ *be the 2-partition set of T*_1_, *and let Q*_2_ *be the 2-partition set of T*_2_. *Then Q*_1_ ⊆ *Q*_2_.

*Proof*. A refinement step adds one edge to *T*_1_ and one 2-partition. By induction on the number of refinement steps to go from *T*_1_ to *T*_2_, we obtain *Q*_1_ ⊆ *Q*_2_.

**Theorem 9**. *If S-tree T*_2_ *can be obtained from S-tree T*_1_ *using a number of refinement steps, then T*_1_ *and T*_2_ *are non-contradictory*.

*Proof*. Let *T*_1_ be an *S*-tree, and let *T*_2_ be a refinement of *T*_1_. Let *Q*_1_ be the set of 2-partitions of *T*_1_, and let *Q*_2_ be the set of 2-partitions of *T*_2_. By Lemma 2, *Q*_1_ ⊆ *Q*_2_. By Lemma 1, *Q*_2_ is not contradictory with itself. Then *Q*_1_ and *Q*_2_ are non-contradictory, since otherwise *Q*_2_ would be contradictory with itself. By definition, *T*_1_ and *T*_2_ are non-contradictory.

The posets given for each gene are used in the construction of one tree for each gene. These trees can contain contradictory information, as illustrated in Fig 24. To be able to identify HGT events, contradictory trees must be identified. This can be done by examining the number of ways leaves and the root in a tree can be partitioned. This is done by examining the cuts in edges that are not incident to leaf nodes. If two trees are contradictory, then there is evidence for HGT.

The *minimum common refinement* of two non-contradictory *S*-trees *T*_1_ and *T*_2_ is an *S*-tree *T*_3_ that is a common refinement of *T*_1_ and *T*_2_ such that any other common refinement of *T*_1_ and *T*_2_ is a refinement of *T*_3_.

**Theorem 10**. *Let T*_1_ *and T*_2_ *be S-trees that are non-contradictory. Let Q*_1_ *and Q*_2_ *be their respective sets of 2-partitions. Then there exists a unique tree T*_3_ *that is their minimum common refinement. Furthermore, if Q*_3_ *is the set of 2-partitions of T*_3_, *then Q*_3_ = *Q*_1_ ∪ *Q*_2_.

*Proof*. Define *Q*_3_ = *Q*_1_ ∪ *Q*_2_. Therefore, *Q*_3_ contains 2-partitions, where each 2-partition is obtained by cutting one edge of the tree *T*_3_. Hence, the set *Q*_3_ can be used to construct the tree *T*_3_, by checking each 2-partition, starting with the 2-partition of minimum cardinality. Siblings in *T*_3_ are inferred and the set is reduced. This process is repeated until only 2-partitions with one of its elements having cardinality one are remaining. Since *Q*_3_ = *Q*_1_ ∪ *Q*_2_ and since *Q*_1_ already corresponds to a tree and also *Q*_2_ corresponds to a tree, all the 2-partitions in *Q*_1_ and *Q*_2_ already correspond to edges in a tree. Therefore, using the two sets, a more refined tree can be constructed. Since *Q*_1_ and *Q*_2_ both contain non-contradictory partitions, and since *Q*_3_ = *Q*_1_ ∪ *Q*_2_, *Q*_3_ also contains non-contradictory partitions, and hence, there exists a tree *T*_3_ that corresponds to *Q*_3_. Using induction, we start by *Q*_1_ and *T*_1_ and add 2-partitions from *Q*_2_ to *Q*_1_. Let *k* be the number of 2-partitions added. If *k* = 1, then a 2-partition is added from *Q*_2_ to *Q*_1_. Since *T*_1_ and *T*_2_ are non-contradictory, a 2-partition that exists in *Q*_2_ but not in *Q*_1_ only adds an internal node and an edge to *T*_1_. Therefore, *T*_1_ becomes a more refined tree. Hence, adding *k* 2-partitions to *T*_1_ will further refine *T*_1_ by adding more edges and internal nodes. Therefore, given *Q*_3_, a set of non-contradictory 2-partitions, a tree *T*_3_ can be constructed.

**Fig 25:**
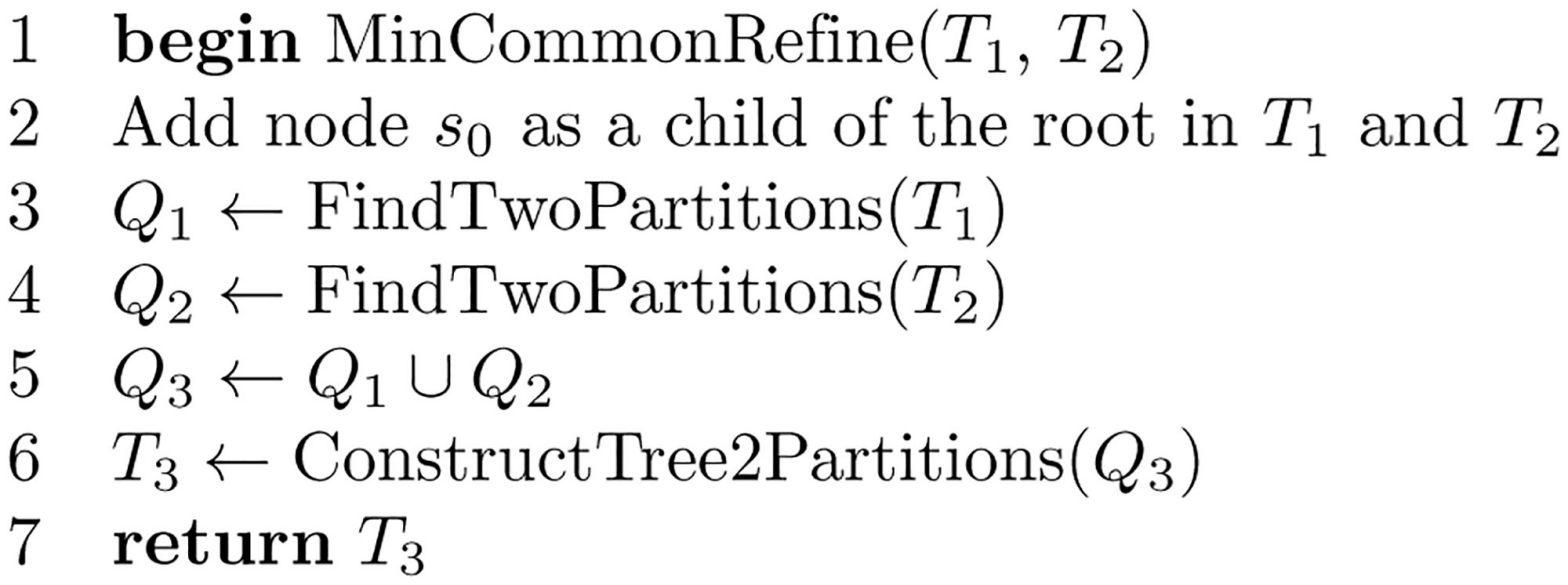
Algorithm to find the minimum common refinement of two trees.

An algorithm for finding the minimum common refinement of *T*_1_ and *T*_2_ is shown in Fig 25. The algorithm finds all 2-partitions of *T*_1_ and *T*_2_. A 2-partition is found by cutting an edge of the tree and finding the leaves in the two subtrees induced. For example, cutting an edge (*i*, *j*), induces two subtrees, one with the root *i* and the other with the root *j*. Performing a depth-first search on the two subtrees finds the leaves in both subtrees. The species set for each subtree composes one of the 2-partitions; therefore, *S*(*i*) composes one partition, and *S*(*j*) composes the other.

**Fig 26:**
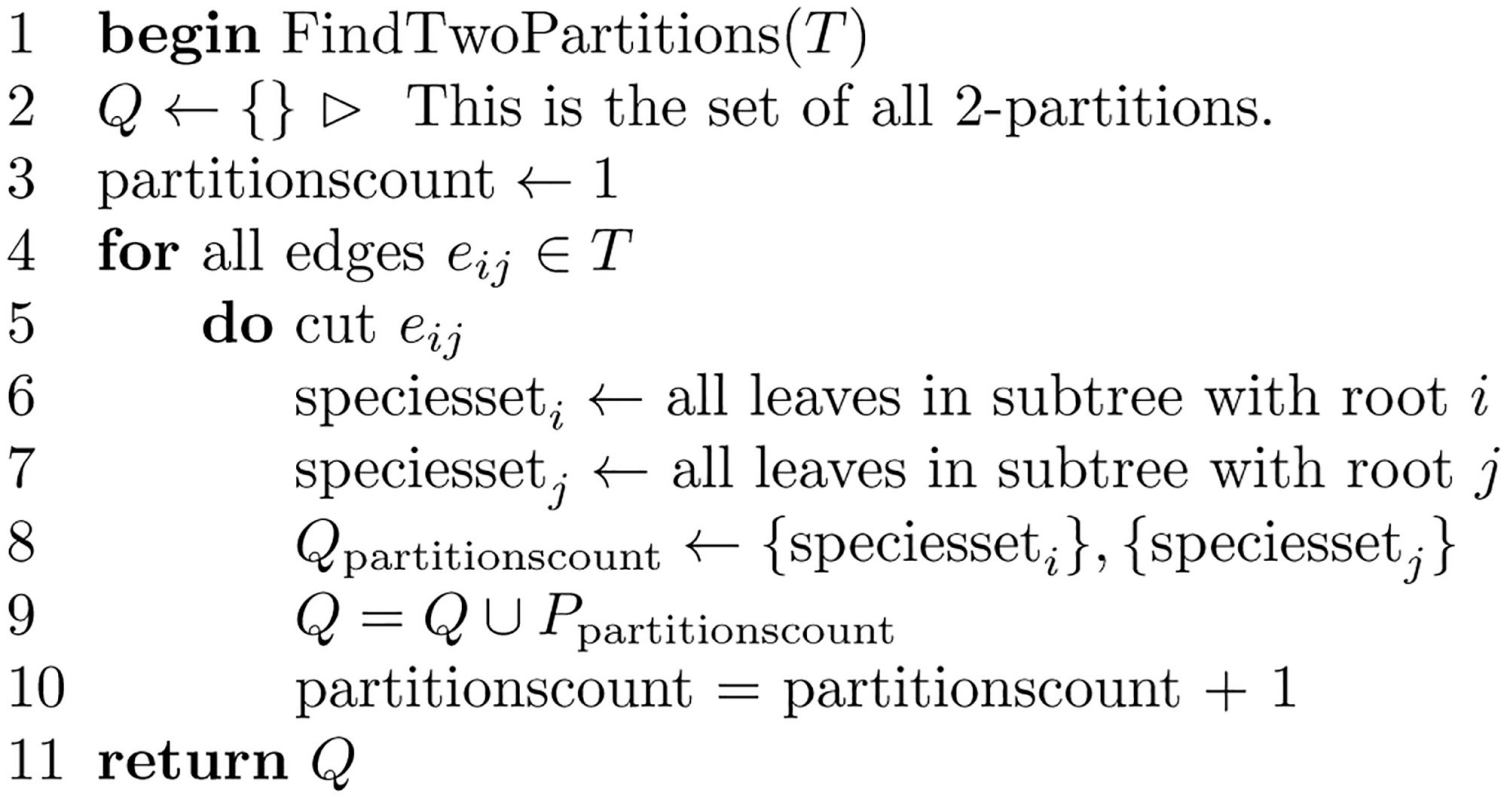
Algorithm to find the 2-partitions set of a given tree.

The subroutine FindTwoPartitions shown in Fig 26 finds the 2-partition set for a given tree. When the 2-partitions sets are found for both trees, a union is performed on these sets to obtain the minimum common refinement tree.

**Fig 27:**
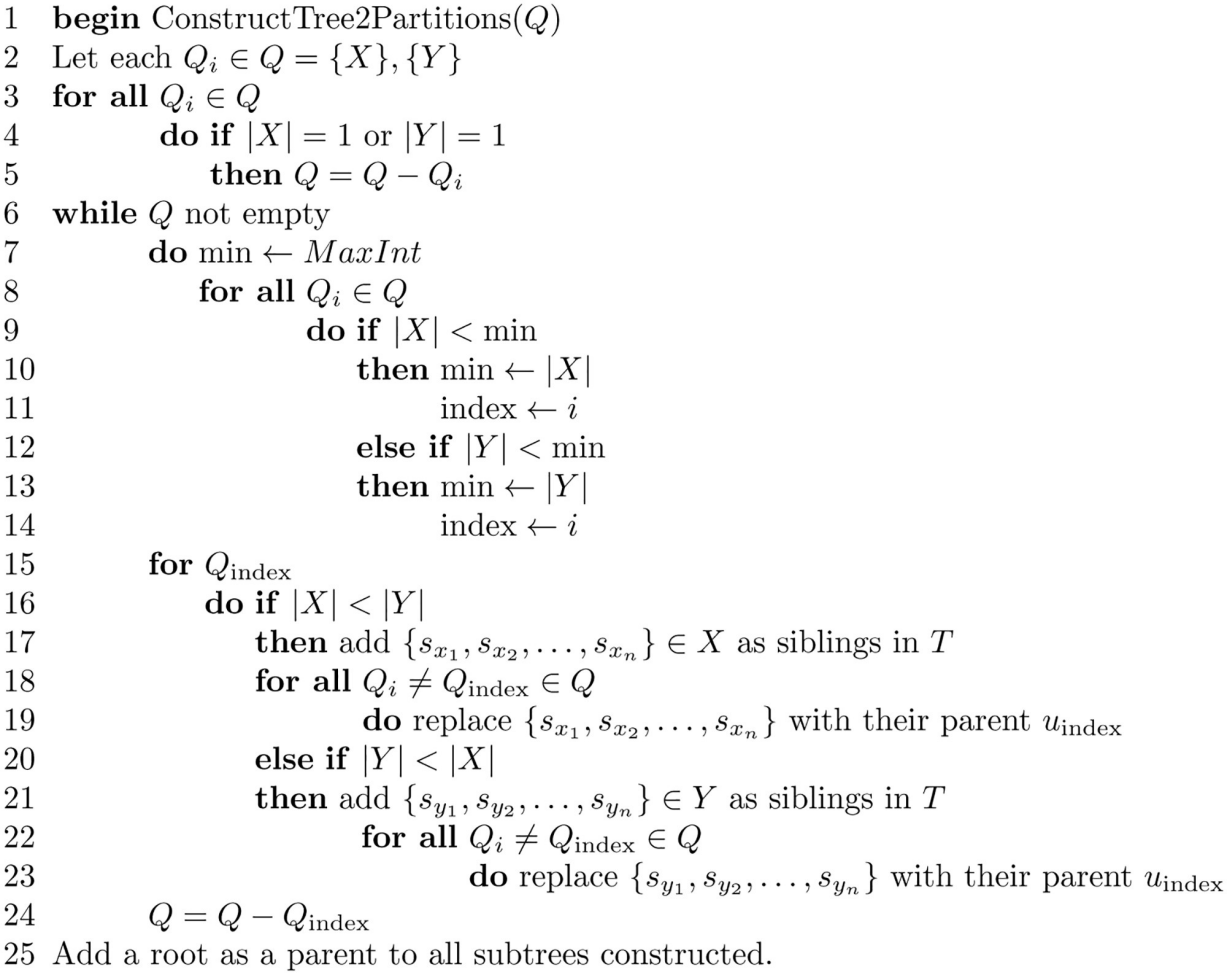
Algorithm to construct a tree from its 2-partitions set.

The algorithm that constructs a tree from its two-partition set is shown in Fig 27, followed by an illustrative example.

**Fig 28:**
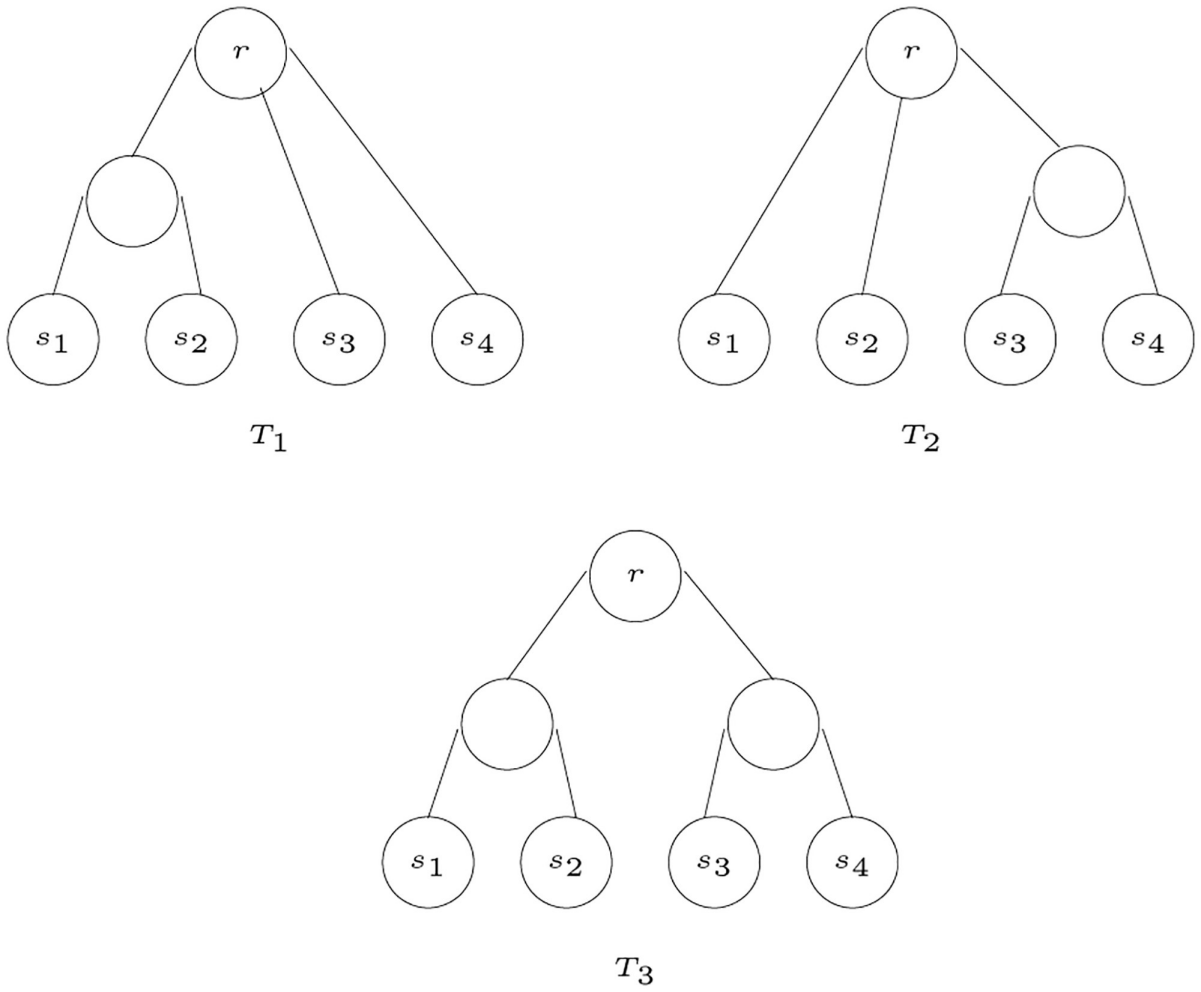
Refinement of *T*_1_ and *T*_2_ into *T*_3_.

An example to show the minimum common refinement, given two *S*-trees, *T*_1_ and *T*_2_, if using a number of refinement steps both trees can be refined into a third *S*-tree *T*_3_, then it is guaranteed that both trees carry non-contradictory information. For example, the two *S*-trees, *T*_1_ and *T*_2_ shown in Fig 28 are non-contradictory and they are both refined into *T*_3_. In this example, *T*_3_ is obtained using the minimum number of refinement steps, hence, *T*_3_ is the minimum common refinement of *T*_1_ and *T*_2_.

**Fig 29:**
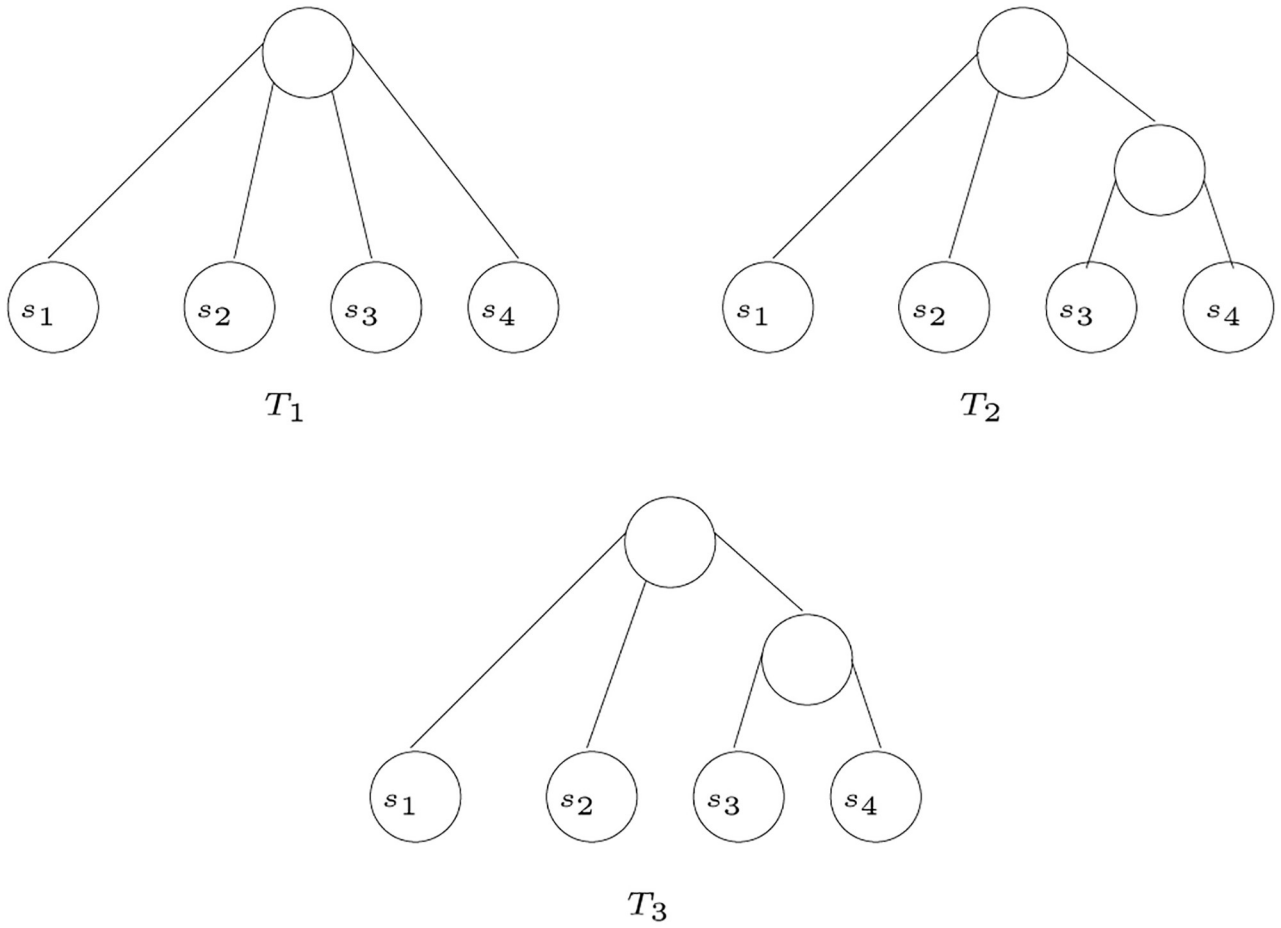
*T*_3_ is the minimum common refinement of *T*_1_ and *T*_2_.

Fig 29 shows an example to illustrate minimum common refinement, where the tree *T*_3_ is the minimum common refinement of the two trees *T*_1_ and *T*_2_, where *T*_3_ is obtained using one refinement step, this refinement step is performed on *T*_1_ by adding a parent for *s*_3_ and *s*_4_. The refined tree is the same tree as *T*_2_.

**Fig 30:**
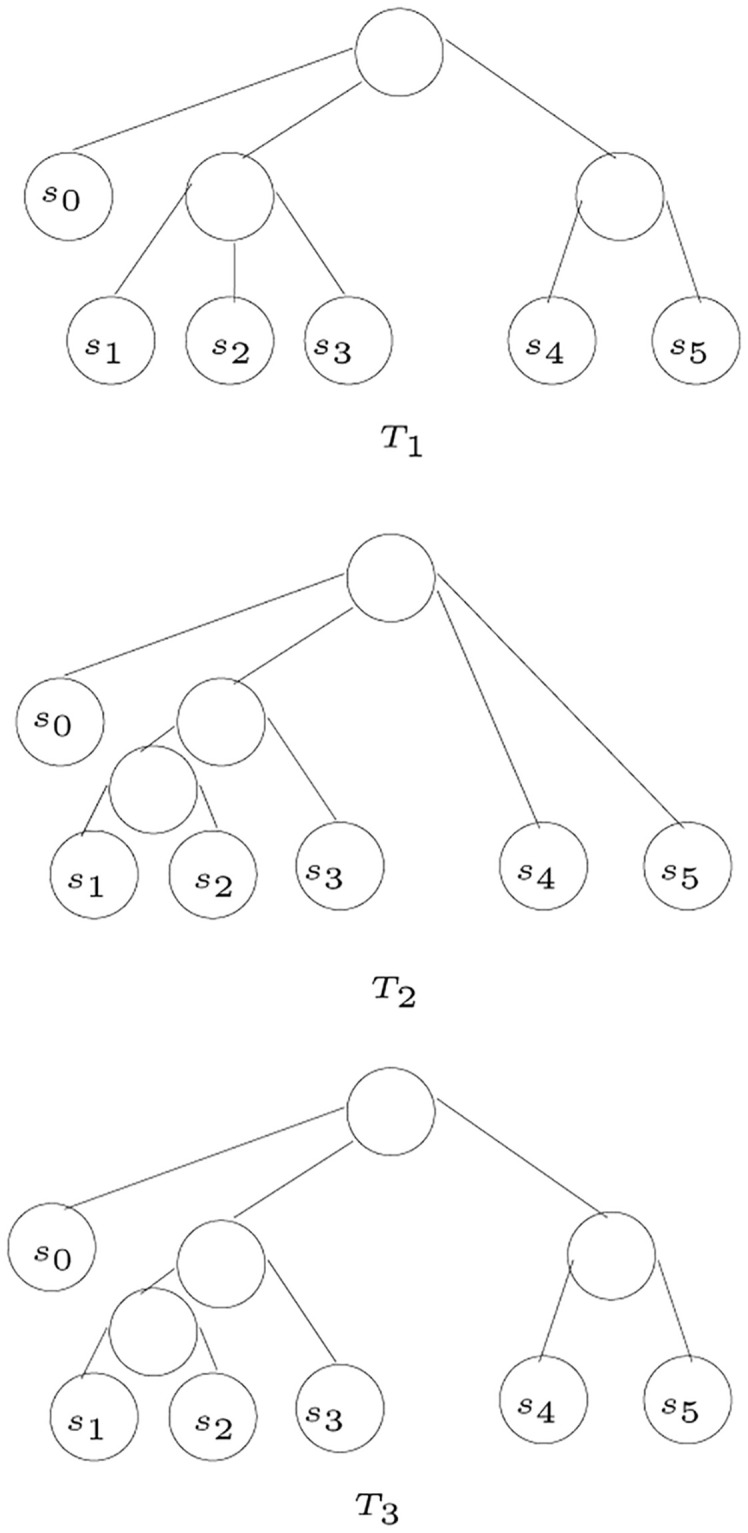
An example to illustrate the algorithm MinCommonRefine.

Fig 30 shows an example to illustrate the algorithm. The node *s*_0_ is added under the root to avoid having equivalent sets for a 2-partition, as these equivalent sets disappear when performing the union operation. In the example, *T*_1_ has eight edges, including the edge connecting the *s*_0_ to the root. Hence, there are eight 2-partitions sets for *T*_1_. Similarly, their are eight 2-partitions sets for *T*_2_. The 2-partitions sets for *T*_1_ are as follows:

$$\begin{array}{ccc} Q_{1} & = & \left\{s_{0}\right\},\left\{s_{1},s_{2},s_{3},s_{4},s_{5}\right\}\\ Q_{2} & = & \left\{s_{1}\right\},\left\{s_{0},s_{2},s_{3},s_{4},s_{5}\right\}\\ Q_{3} & = & \left\{s_{2}\right\},\left\{s_{0},s_{1},s_{3},s_{4},s_{5}\right\}\\ Q_{4} & = & \left\{s_{3}\right\},\left\{s_{0},s_{1},s_{2},s_{4},s_{5}\right\}\\ Q_{5} & = & \left\{s_{4}\right\},\left\{s_{0},s_{1},s_{2},s_{3},s_{5}\right\}\\ Q_{6} & = & \left\{s_{5}\right\},\left\{s_{0},s_{1},s_{2},s_{3},s_{4}\right\}\\ Q_{7} & = & \left\{s_{1},s_{2},s_{3}\right\},\left\{s_{0},s_{4},s_{5}\right\}\\ Q_{8} & = & \left\{s_{4},s_{5}\right\},\left\{s_{0},s_{1},s_{2},s_{3}\right\} \end{array}$$

The 2-partitions sets for *T*_2_ are as follows:

$$\begin{array}{ccc} Q_{1} & = & \left\{s_{0}\right\},\left\{s_{1},s_{2},s_{3},s_{4},s_{5}\right\}\\ Q_{2} & = & \left\{s_{1}\right\},\left\{s_{0},s_{2},s_{3},s_{4},s_{5}\right\}\\ Q_{3} & = & \left\{s_{2}\right\},\left\{s_{0},s_{1},s_{3},s_{4},s_{5}\right\}\\ Q_{4} & = & \left\{s_{3}\right\},\left\{s_{0},s_{1},s_{2},s_{4},s_{5}\right\}\\ Q_{5} & = & \left\{s_{4}\right\},\left\{s_{0},s_{1},s_{2},s_{3},s_{5}\right\}\\ Q_{6} & = & \left\{s_{5}\right\},\left\{s_{0},s_{1},s_{2},s_{3},s_{4}\right\}\\ Q_{7} & = & \left\{s_{1},s_{2}\right\},\left\{s_{0},s_{3},s_{4},s_{5}\right\}\\ Q_{8} & = & \left\{s_{1},s_{2},s_{3}\right\},\left\{s_{0},s_{4},s_{5}\right\} \end{array}$$

The union of the two sets of partitions gives the following 2-partitions sets, which are the sets that give the tree *T*_3_:

$$\begin{array}{ccc} Q_{1} & = & \left\{s_{0}\right\},\left\{s_{1},s_{2},s_{3},s_{4},s_{5}\right\}\\ Q_{2} & = & \left\{s_{1}\right\},\left\{s_{0},s_{2},s_{3},s_{4},s_{5}\right\}\\ Q_{3} & = & \left\{s_{2}\right\},\left\{s_{0},s_{1},s_{3},s_{4},s_{5}\right\}\\ Q_{4} & = & \left\{s_{3}\right\},\left\{s_{0},s_{1},s_{2},s_{4},s_{5}\right\}\\ Q_{5} & = & \left\{s_{4}\right\},\left\{s_{0},s_{1},s_{2},s_{3},s_{5}\right\}\\ Q_{6} & = & \left\{s_{5}\right\},\left\{s_{0},s_{1},s_{2},s_{3},s_{4}\right\}\\ Q_{7} & = & \left\{s_{1},s_{2}\right\},\left\{s_{0},s_{3},s_{4},s_{5}\right\}\\ Q_{8} & = & \left\{s_{1},s_{2},s_{3}\right\},\left\{s_{0},s_{4},s_{5}\right\}\\ Q_{9} & = & \left\{s_{0},s_{1},s_{2},s_{3}\right\},\left\{s_{4},s_{5}\right\} \end{array}$$

Lets consider the following two-partition set, *Q*, to illustrate the algorithm.

$$\begin{array}{ccc} Q_{1} & = & \left\{s_{0}\right\},\left\{s_{1},s_{2},s_{3},s_{4},s_{5}\right\}\\ Q_{2} & = & \left\{s_{1}\right\},\left\{s_{0},s_{2},s_{3},s_{4},s_{5}\right\}\\ Q_{3} & = & \left\{s_{2}\right\},\left\{s_{0},s_{1},s_{3},s_{4},s_{5}\right\}\\ Q_{4} & = & \left\{s_{3}\right\},\left\{s_{0},s_{1},s_{2},s_{4},s_{5}\right\}\\ Q_{5} & = & \left\{s_{4}\right\},\left\{s_{0},s_{1},s_{2},s_{3},s_{5}\right\}\\ Q_{6} & = & \left\{s_{5}\right\},\left\{s_{0},s_{1},s_{2},s_{3},s_{4}\right\}\\ Q_{7} & = & \left\{s_{1},s_{2}\right\},\left\{s_{0},s_{3},s_{4},s_{5}\right\}\\ Q_{8} & = & \left\{s_{1},s_{2},s_{3}\right\},\left\{s_{0},s_{4},s_{5}\right\}\\ Q_{9} & = & \left\{s_{0},s_{1},s_{2},s_{3}\right\},\left\{s_{4},s_{5}\right\} \end{array}$$

The algorithm starts by removing all sets with cardinality 1. So the set *Q* is reduced to the following:

$$\begin{array}{ccc} Q_{7} & = & \left\{s_{1},s_{2}\right\},\left\{s_{0},s_{3},s_{4},s_{5}\right\}\\ Q_{8} & = & \left\{s_{1},s_{2},s_{3}\right\},\left\{s_{0},s_{4},s_{5}\right\}\\ Q_{9} & = & \left\{s_{0},s_{1},s_{2},s_{3}\right\},\left\{s_{4},s_{5}\right\} \end{array}$$

The set with the minimum cardinality is in *Q*_7_, therefore, the species *s*_1_ and *s*_2_ are detected as siblings and they are replaced by a parent node in all sets. Therefore, *Q* is modified to the following:

$$\begin{array}{ccc} Q_{8} & = & \left\{u_{1},s_{3}\right\},\left\{s_{0},s_{4},s_{5}\right\}\\ Q_{9} & = & \left\{s_{0},u_{1},s_{3}\right\},\left\{s_{4},s_{5}\right\} \end{array}$$

**Fig 31:**
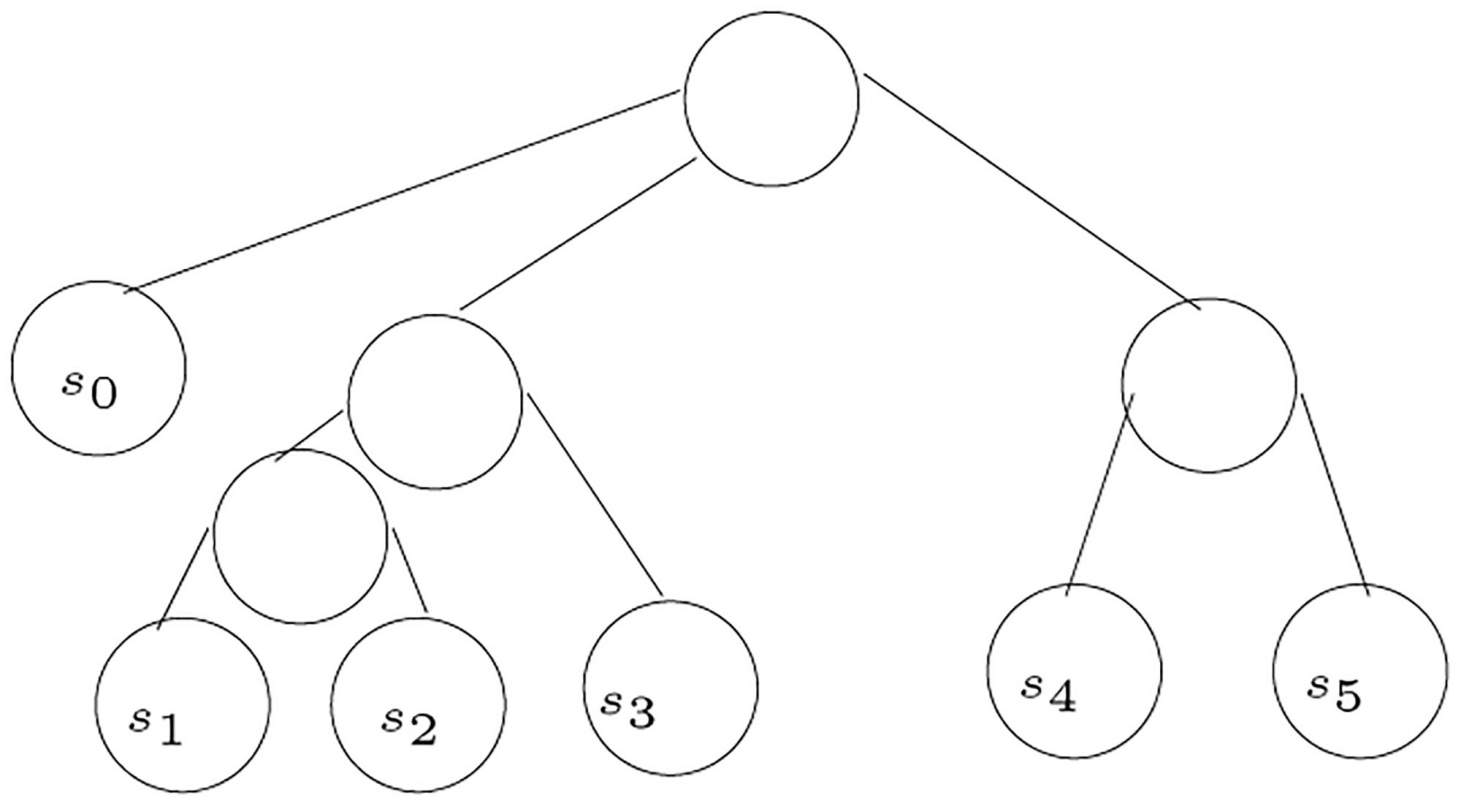
An example to illustrate the algorithm ConstructTree2Partitions.

The next step finds the minimum cardinality in both *Q*_8_ and *Q*_9_, where *u*_1_ and *s*_3_ are siblings, and *s*_4_ and *s*_5_ are siblings. When *Q*_8_ and *Q*_9_ are removed from *Q*, it becomes empty and the root connects the subtrees constructed. Fig 31 shows the tree constructed from the two-partition set *Q*.

**Theorem 11**. *The time complexity of MinCommonRefine is* *O*(*mn* + *n*^2^).

*Proof*. Let *n* be the number of species. Let *m* be the number of edges in a tree *T*. The subroutine FindTwoPartitions on Lines 3 and 4 is *O*(*mn*) Line 5 performs a union operation linear in the number of species. Line 6 constructs the tree from its two-partition set, ConstructTree2Partitions is *O*(*n*^2^). Therefore, the overall complexity of the algorithm MinCommonRefine is *O*(*mn* + *n*^2^).

## Inferring HGT from posets

In this section, we show how posets and trees are used to infer HGT.

The problem is defined as follows:

Inferring HGT From Posets
INSTANCE: Set *S* = {*s*_1_, *s*_2_, …, *s*_*n*_} of *n* taxa; set *G* = {*g*_1_, *g*_2_, …, *g*_*m*_} of *m* genes; *mn* individual posets *P*_*ij*_ = (*S*, <_*ij*_), for 1 ≤ *i* ≤ *m* and 1 ≤ *j* ≤ *n*.
SOLUTION: Sets of genes corresponding to contradictory trees.

**Fig 32:**
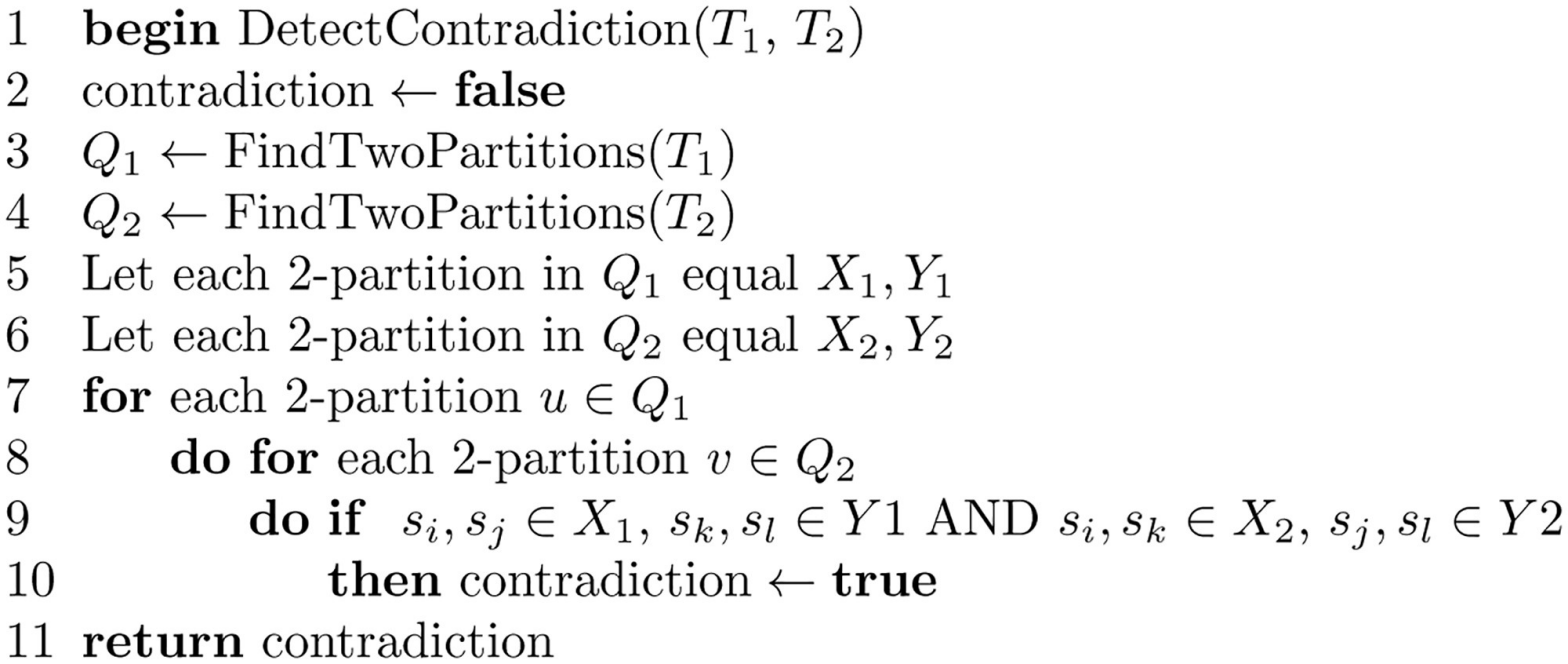
Algorithm to detect contradiction between two trees.

A number of steps are followed to be able to infer HGT events. First, trees are constructed from posets, then the different trees are compared, where contradictory trees are identified. Trees that are contradictory with the majority of trees suggest HGT. Other events such as gene duplication, gene loss, and incomplete lineage sorting can cause the incongruence of trees [40]. In the “Constructing an *S*-tree From a Set of Posets” Section, we show how trees are constructed from posets; in what follows, we show how contradictory trees are detected. The algorithm DetectContradiction shown in Fig 32 takes two trees as input and detects whether they are contradictory or not.

The process of identifying which genes are candidates of HGT proceeds as follows. Two *S*-trees *T*_1_ and *T*_2_ are tested for contradiction. If they are contradictory, then they belong to two different sets, if not then they are placed in one set. The process continues. If the next tree to be tested is *T*_3_, then it is compared with one tree from each set to test to which set the tree *T*_3_ belongs. It is expected that the majority of the trees will be non-contradictory, with some trees contradicting this majority, so there will be one set with a higher cardinality. Therefore, the other sets, which are the minority, are considered candidates for HGT.

The algorithm performs ideally when all the trees are completely refined (binary) trees, where the trees that are not identical are considered contradictory. In what follows, some real life HGT examples are shown to support the argument that the genes involved in HGT are a minority and that there will always be a dominant tree. In Ponting [41], it is indicated that only 0.5% of all human genes were copied into the genome from bacteria by HGT. Rujan and Martin [42] analyzed how many genes in *Arabidopsis* come from cyanobacteria, They used a sample of 3961 *Arabidopsis* nuclear protein-coding genes and compared those with the complete set of proteins from yeast and 17 reference prokaryotic genomes, including one cyanobacterium. In their analysis of 386 phylogenetic trees, they found that the number of genes horizontally transferred to *Arabidopsis* from *cyanobacteria* falls between approximately 400 genes and approximately 2200 genes. That is between 1.6% and 9.2% of nuclear genes.

**Fig 33:**
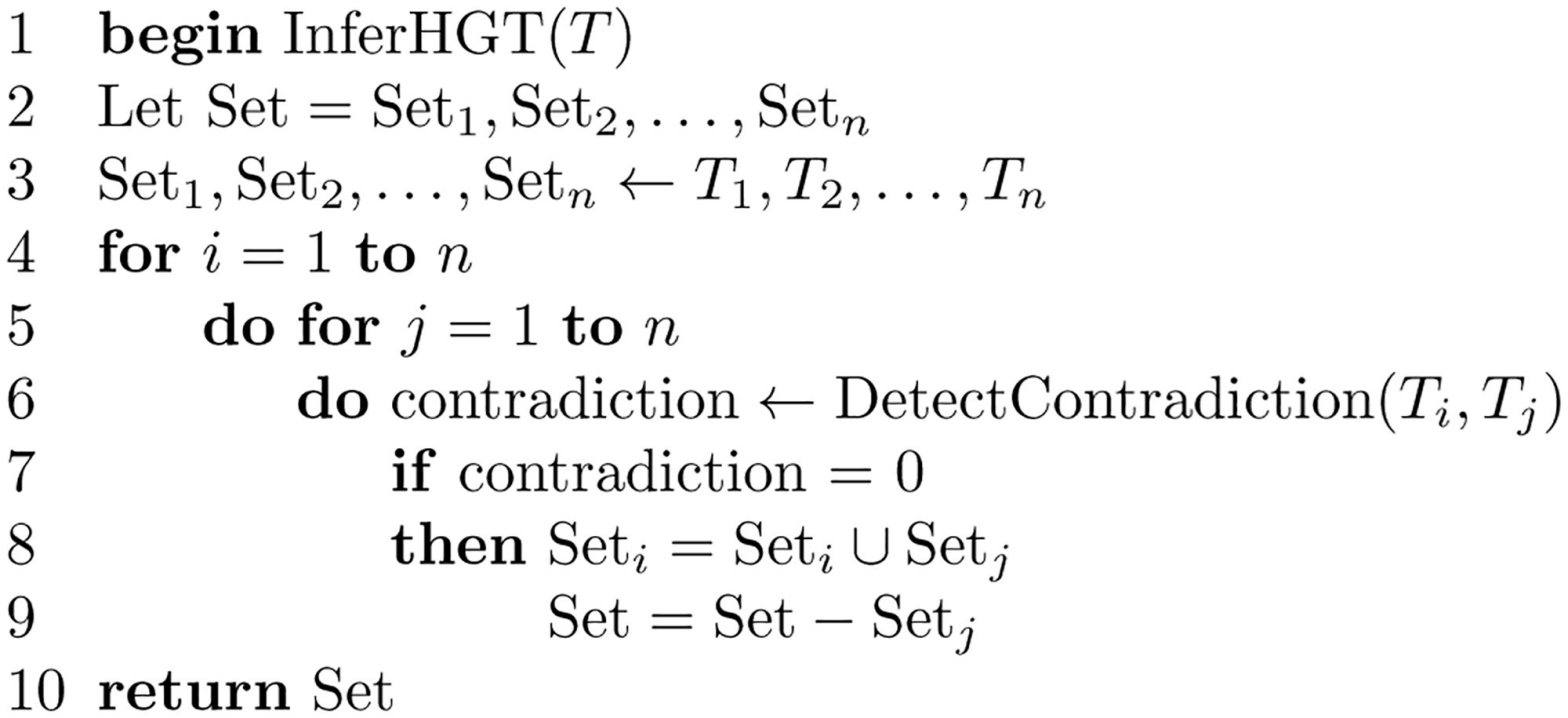
Algorithm to infer HGT.

The algorithm InferHGT is shown in Fig 33. The input to the algorithm is a set of trees *T* = {*T*_1_, *T*_2_, …, *T*_*n*_}, where *n* is the number of trees and also the number of genes.

**Fig 34:**
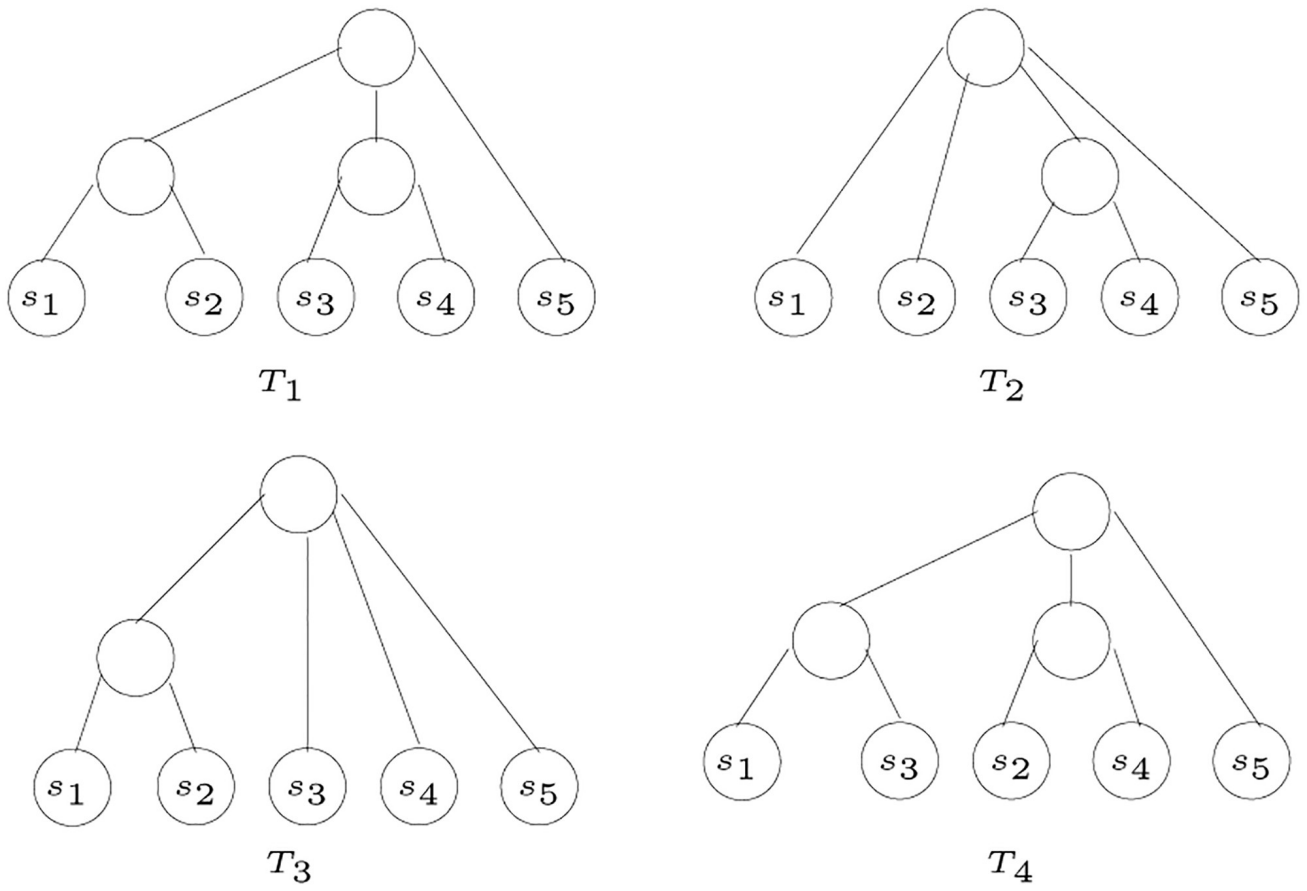
An example to illustrate the algorithm InferHGT.

An example to illustrate the algorithm for inferring HGT is shown in Fig 34, where the trees *T*_1_, *T*_2_, and *T*_3_ are non-contradictory, while the tree *T*_4_ contradicts the three trees. In *T*_4_ there is a 2-partition that places the two species {*s*_1_, *s*_3_} in one partition, and {*s*_2_, *s*_4_} in another partition. This 2-partition contradicts the other three trees. Therefore, the gene corresponding to *T*_4_ is a candidate of HGT, where a horizontal transfer occurred between *s*_1_ and *s*_3_, or *s*_2_ and *s*_4_. The network in Fig 1 shows the possible horizontal transfers. We note that the figure documents both the existence of two possible horizontal transfers but also their directionality, which is especially valuable for any further investigation.

**Theorem 12**. *InferHGT has complexity max*(*O*(*n*^2^), *O*(*m*^2^*n*)).

*Proof*. The two nested loops on lines 4 and 5 are *O*(*n*^2^), where *n* is the number of trees. The subroutine DetectContradiction on line 6 is *O*(*m*^2^*n*), where *m* is the number of edges in a tree.

## Conclusions

We have introduced the theoretical problem of inferring HGT using partial orders, where there is one poset per gene per species. These posets have been used to construct *S*-trees for the genes corresponding to these posets, one tree for each gene. These trees are then compared, where the trees that contradict the majority of trees correspond to genes that are candidates for HGT. An algorithm for identifying contradiction is presented and then used in the algorithm to infer HGT. The concept of refinement is also presented in this paper, where it can also be used to identify contradiction among trees. An algorithm for finding a minimum common refinement for two trees is also presented. This algorithm finds the union of the 2-partition sets of two trees and then uses this set to construct a third tree, which is their minimum common refinement. Other points can be further studied in this problem. For example, more effort could be done to find solutions to the problem of incorrect or missing data in the input posets. This will be incredibly challenging, but, from a practical viewpoint, it would be most valuable. Another point is to develop algorithms that use the refinement of trees for identifying contradictory trees, where two contradictory trees do not have a common refinement.
